# RAId_aPS: MS/MS Analysis with Multiple Scoring Functions and Spectrum-Specific Statistics

**DOI:** 10.1371/journal.pone.0015438

**Published:** 2010-11-16

**Authors:** Gelio Alves, Aleksey Y. Ogurtsov, Yi-Kuo Yu

**Affiliations:** National Center for Biotechnology Information, National Library of Medicine, National Institutes of Health, Bethesda, Maryland, United States of America; University of South Florida College of Medicine, United States of America

## Abstract

Statistically meaningful comparison/combination of peptide identification results from various search methods is impeded by the lack of a universal statistical standard. Providing an 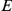-value calibration protocol, we demonstrated earlier the feasibility of translating either the score or heuristic 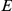-value reported by any method into the textbook-defined 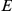-value, which may serve as the universal statistical standard. This protocol, although robust, may lose spectrum-specific statistics and might require a new calibration when changes in experimental setup occur. To mitigate these issues, we developed a new MS/MS search tool, RAId_aPS, that is able to provide *spectrum-specific* 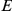-values for *additive* scoring functions. Given a selection of scoring functions out of RAId score, K-score, Hyperscore and XCorr, RAId_aPS generates the corresponding score histograms of *all possible* peptides using dynamic programming. Using these score histograms to assign 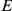-values enables a calibration-free protocol for accurate significance assignment for each scoring function. RAId_aPS features four different modes: (i) compute the total number of possible peptides for a given molecular mass range, (ii) generate the score histogram given a MS/MS spectrum and a scoring function, (iii) reassign 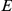-values for a list of candidate peptides given a MS/MS spectrum and the scoring functions chosen, and (iv) perform database searches using selected scoring functions. In modes (iii) and (iv), RAId_aPS is also capable of combining results from different scoring functions using spectrum-specific statistics. The web link is http://www.ncbi.nlm.nih.gov/CBBresearch/Yu/raid_aps/index.html. Relevant binaries for Linux, Windows, and Mac OS X are available from the same page.

## Introduction

### General Background

Gaining popularity in biology over the last decade, mass spectrometry (MS) has become the core technology in the field of proteomics. Although this technology holds the promise to identity and quantify proteins in complex biological mixtures/samples, such a goal has not yet been achieved due to the presence of a number of difficulties ranging from experimental design and experimental protocol standardization to data analysis [1]–[3]. This paper mainly focuses on the data analysis, especially providing accurate statistical significance assignments for peptide candidates in peptide identifications. There are many peptide identification methods that are available to the proteomics community. Because different identification methods process (filter) the MS/MS spectra differently and also have different scoring functions, it is natural for users to wish to compare search results from different search methods or to combine these results to enhance identification confidence. Nevertheless, there are important issues to be addressed prior to successfully reaching this goal.

Due to intrinsic experimental variability, differences in the peptide chemistry, peptide-peptide interactions, ionization sources, and mass analyzers used, it is natural to expect among tandem mass spectra variations in signal to noise ratios even when each peptide in the mixture has equal molar concentration. That said, one anticipates the noise in a mass spectrum to be spectrum-specific and the meaning of a search score depends on its context, *i.e.*, the spectrum used. That is, although search score can be used to compare candidate peptides associated with the same query spectrum, it is no longer a valid measure when one wishes to compare peptides identified across spectra. Not only posing a challenge for ranking identified peptides within a single experiment, this also raise a serious problem when one wishes to compare or combine search results from different scoring functions (or search methods).

If one knows how to *translate* the score or reported 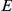-value of one method to that of another method, or to a universal standard, it helps significantly the task of comparing/combining search results. This is particularly true when one wishes to combine search results from multiple scoring functions. We showed in an earlier publication [4] that it is possible to use the textbook-defined 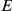-value as that universal standard. Providing an 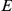-value calibration protocol, we demonstrated the feasibility of translating either the score or heuristic 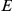-value reported by any method to the textbook-defined 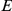-value, the proposed universal statistical standard. This protocol, although robust, may (a) lose spectrum-specific statistics, and may (b) require a new calibration when changes in experimental set up occur.

Without attempting a universal statistical standard, several machine-learning based approaches have been developed to either re-rank identified candidate peptides [5], [6] or to combine search results from several search methods [7], [8]. These approaches require for their analyses training data set(s), either pre-constructed or obtained on-the-fly, to aid the parameter selections for their discriminant functions. For methods with feature vector (allowed to contain some spectrum-specific quantities) updated on-the-fly [6], [8], the spectrum-specific bias may be partially compensated, but not giving rise to spectrum-specific statistics. This is because the feature vector, although may be trained with spectrum-specific quantities, aims to categorize the whole training set into finite number of classes but does not solely reflect the properties of any individual spectrum.

To address the issue of spectrum-specific statistics, we developed a new MS/MS search tool, RAId_aPS (a new module of the RAId suite), that is able to provide *spectrum-specific* 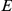-values for additive scoring functions that do not have known theoretical score distributions. RAId_aPS provides the users with four different modes to choose from: (i) compute the total number of possible peptides (TNPP), (ii) generate score histogram, (iii) reassign 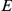-values, and (iv) database search. In modes (iii) and (iv), RAId_aPS is also capable of combining results [9] from different scoring functions. Founded on the algorithm published earlier [10], mode (i) is a straight implementation of an existing idea. However, modes (ii) to (iv) are novel, albeit at different levels. Mode (ii) uses the algorithm published earlier [10], nevertheless, generating the *all-possible*-peptide (APP) score histograms of different scoring functions was never done. Mode (iii) is novel from the concept to its implementation. Modes (i–iii) do not have counter-parts in other components of RAId suite. Mode (iv) is similar to RAId_DbS [11] in the sense that it performs database searches. However, the difference between mode (iv) of RAId_aPS and RAId_DbS lies in the use of statistics. The theoretical score distribution of RAId_DbS fits score histogram of database peptides per spectrum, while mode (iv) RAId_aPS uses score distributions of APP and is able to provide statistics for multiple scoring functions.

The term “all possible peptides” (or APP) deserves some deliberation. The pool of APP includes any linear arrangement of amino acids. Therefore, when considering peptides of 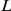 amino acids without modification, the APP pool includes all the 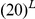 combinations. For the purpose of mass spectrometry data analysis, instead of peptides with a fixed length one is more interested in APP within a specified molecular mass range. The number of possible peptides (PP) within a molecular mass range is much larger than the number of database peptides within the same molecular mass range. For example, for the molecular mass range 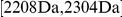, there are approximately 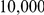 peptides in the *Bos Taurus* database, while there are in total 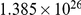 PP with lengths (number of amino acids) ranging from 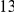 to 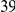.

Using dynamic programming, RAId_aPS generates the score histograms from scoring APP. These score histograms are then used to assign accurate, spectrum-specific 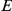-values. Since RAId_aPS uses the score histograms, or the (weighted) rank of each candidate peptide considered among APP, it is already in conformity to the textbook defined 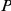-value and thus there is no need to *translate* the score or heuristic 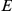-value into the universal standard. Consequently, RAId_aPS is able to provide a calibration-free protocol for accurate significance assignment and for combining search results.

In order to provide a clear exposition, it is necessary for us to go into some technical details. Readers not interested in the details, however, may want to read the results section first and then come back to read other sections. To make the paper easier to read and more modular, we outline below the organization of this paper. In the Technical Background subsection below, we will review the similarities and differences between two major approaches in dealing with peptide identification statistics, describe how one may achieve calibration-free, spectrum-specific statistics. In the Method section, we first describe the dynamic programming algorithm needed to generate the score distribution of APP, followed by spectral filtering procedures each associated with a scoring function implemented. The incorporation of the four scoring functions are then reported since some of them are nontrivial to encode via dynamic programming. We then describe how the APP statistics are implemented in practice, how to include modified amino acids in APP statistics, and how to combine search results from different scoring functions. In the Results section, we describe several tests performed using various modes of RAId_aPS, as well as the 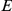-value accuracy assessment. The paper is then concluded by the Discussion section. All the technical aspects that are not most essential in understanding the basic idea are provided either as supplementary texts or supplementary figures. The most important message is that RAId_aPS serves as a calibration-free, statistically sound method for comparing or combining search results from different scoring functions.

### Technical Background

Since this paper is focused on the statistical aspect of peptide identifications, we will start with such an example. In general, it is rather easy to rank candidate peptides given a tandem mass spectrum. Once a scoring function is selected to score peptides, *qualified* database peptides (those within a molecular mass range and with correct enzymatic cleavages) can be ranked based on their scores. However, it becomes difficult to rank candidate peptides across all spectra. Although a number of publications have proposed different ways tailored to deal with various aspects of this difficulty [4], [12], this problem remains very challenging. Should one take the best candidate peptide per spectrum and then postprocess to globally re-rank those best hits or should one devise something different to achieve the maximum robustness? Instead of discussing the differences between these two possibilities, we first wish to point out a common theme that is often unnoticed: spectrum-specificity.

### Spectrum Specificity

As mentioned in the Introduction section, spectrum-specificity has not been emphasized enough. However, there does exist evidence of community's recognition of this point. For example, by picking the best hit out of each spectrum, one is acknowledging spectrum-specificity, because one has chosen to keep the best candidate per spectrum regardless of the fact that the best hit in one spectrum might have lower score than the second best hit in some other spectrum. In other words, by picking only the best hits one has endorsed the view that the score should not be used as an objective measure of identification confidence across *all* candidate peptides; or more precisely, the meaning of score depends on its context, *i.e.*, the spectrum used.

There exists another route to apply the concept of spectrum-specificity. That is to use a spectrum-specific score distribution to assign an 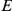-value to each candidate peptide of a spectrum. Although the term spectrum-specific statistics was not explicitly mentioned, the proposal of Fenyo and Beavis [13] to fit per spectrum the tail of score distribution to an exponential represents the first attempt, to the best of our knowledge, in this direction. The concept of spectrum-specific statistics was formally introduced by Alves and Yu [14]. The same group also developed RAId_DbS [11], so far the only database search tool with a theoretically derived spectrum-specific score distribution. The importance of spectrum-specific statistics is then emphasized through a series of publications [4], [9], [11], [15]. The key point of this type of approach is to exemplify spectrum-specificity via spectrum-specific score statistics. After describing the common theme, spectrum specificity, we now turn to features associated with different types of approaches to elucidate the usefulness of an even more general statistical framework.

### Best hit per spectrum versus Accurate 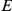-value

When keeping only the best hit per spectrum, a global re-ranking among those best hits becomes necessary in order to decide which best hits to trust over the others. This is usually achieved in one of the two ways to be described. The first possible choice is to use the original score in conjunction with either false discovery rate (FDR) or 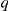-value analysis through introduction of a decoy database. The second choice is to use some kind of *refined* score in conjunction with an empirical expectation-maximization-based Bayesian approach [5]. This global re-ranking type of strategies, unfortunately, makes assumptions contradicting spectrum-specificity, a fundamental fact that is respected when only the best hit per spectrum is retained.

In the FDR (be it global or local) or 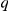-value analyses, one pools together the best hits across spectra and order the hits by their scores. This contradicts the idea of picking best hit per spectrum, which essentially endorses the notion that the meaning of a peptide score is spectrum-dependent and can't be used to rank peptides globally across spectra. For the Bayesian type of analyses [5], one assumes the existence of two score distributions: one for the score of correctly identified spectra, in terms of best hit, and another for the score of incorrectly identified spectra. This means that all correctly identified spectra –in terms of best hit– should be ranked according to the best hit's refined score, implying that one may use the refined score to assign relative identification confidence across spectra. This again contradicts the idea that the meaning of a peptide score is spectrum-dependent. Furthermore, to perform the expectation maximization procedure, one often needs to *assume* the parametric forms of the two distribution functions, which might not be applicable to all scoring functions.

When the reported spectrum-specific 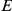-value (assigned to each of the candidate peptides per spectrum) is in agreement with its definition, it can serve as an objective measure of identification confidence. For a given spectrum and a score threshold, the 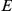-value associated with that score threshold is defined to be the expected number of false hits that have score better than or equal to that threshold. In simple terms, the 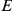-value associated with a candidate peptide in the database may be viewed as the number of false positive hits anticipated, from querying a spectrum, before calling the peptide at hand a true positive hit. However, a previous study [11] showed that most 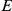-value reporting methods investigated report inaccurate 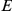-values. To rectify this problem, we provided a protocol [4] to *calibrate* 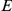-values reported by other search methods, including search tools that don't report 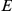-values such as ProbID [16] and SEQUEST [17]. However, the calibration procedure cannot restore/recreate spectrum-specificity for methods not reporting 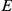-values or reporting 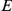-values that are not obtained via characterizing the score histogram for each spectrum (spectrum-specific score modelling).

Nevertheless, spectrum-specific statistics can be obtained provided that one extracts statistical significance from the score histogram for each spectrum [4]. A recent reimplementation [18]–[20] of the SEQUEST XCorr follows exactly this idea. To avoid possible confusion, however, we must first note that the 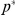-value in reference [18] is actually the 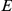-value. Authors of reference [18] *assume* that the XCorr from every spectrum can be fitted by a stretched exponential without providing, like most other methods, a measure on the agreement between the best fitted parametric form and the score distribution per spectrum. To ensure the accuracy of statistics, a measure of the goodness of the model [11], [21] is actually necessary even for scoring systems that have a theoretically characterized distribution. This is because very biased sampling might lead to a discrepancy between the theoretical distribution and the score distribution, not to mention a discrepancy between a fitted parametric form and the score distribution.

One way to circumvent the aforementioned problem is to apply a target-decoy strategy at the *per spectrum* level. This means that one uses the hits from decoy database to estimate the identification confidence of peptides from the target database. This approach, unfortunately, is not computationally efficient because one will need a decoy database that is much larger than the target database in order to have a good estimate of the 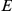-value for each hit in the target database. For example, if the number of qualified peptides in the decoy database is 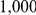 times that in the target database, and if a peptide in the target database scores between the third and the fourth decoy hits, then that peptide will acquire an 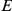-value between 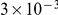 and 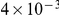. And if there are target hits that score better than the best decoy hit, all one can say is that they all have 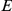-values smaller than 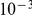. If one keeps increasing the size of the decoy database, one will eventually be able to *globally* rank the candidate peptides from all spectra using 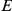-value. However, computational efficiency prevents us from using this strategy.

These aforementioned problems associated with obtaining spectrum-specific statistics can be avoided provided that one uses a search method that has a theoretically derived score distribution [11]. However, restricting to methods that have theoretically derived statistics is not necessarily the best strategy since each search method does have different strengths [9], [22]. It can be advantageous to combine different types of search scores. Therefore, for assigning peptides' identification confidence, it is desirable to have a unified framework which we now turn to.

### APP Statistics (calibration-free)

Alves and Yu in 2005 proposed [14] using the *de novo* rank as the statistical significance measure. Despite the simplicity of this idea, it was never fully carried out. Since it is this idea that inspired the development of RAId_aPS, we need to describe the basic concept to some detail so that various extensions employed in RAId_aPS can be properly explained.

The fundamental idea is as follows. For a given MS/MS spectrum 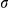 with parent molecular mass 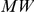 and a given mass error tolerance 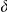, we denote by 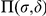 the set of APP subjected to enzymatic cleavage condition in the mass range 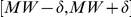. We also denote by 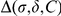 the set of peptides in the (target) database, subjected to a set of conditions 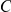, in the mass range 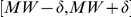. The set of conditions 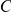 may contain, for example, the enzymatic cleavage constraints, number of miscleavage sites per peptide allowed, and others [23]. The following argument is also applicable to the case when one wishes to weight each peptide in the APP set by its elemental composition. This may be used to form a background model mimicking the amino acid composition in the target database [10], [24].

Let 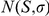 be the (weighted) number of peptides out of 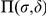 that have scores greater than or equal to 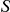. We then define the APP 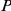-value corresponding to score 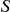 by 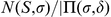, with 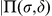 representing the total (weighted) number of peptides in the set 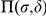. In general, for a given spectrum 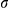 and a score cutoff 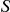, the 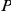-value 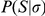 refers to the probability for a *qualified* random peptide to attain a score greater than or equal to 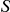 when using spectrum 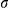 as a query. If a database contains 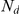 qualified, unrelated random peptides, one will expect to have 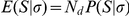 number of random peptides to have quality score greater than or equal to 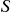. This expectation value 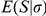 is by definition the 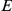-value associated with score cutoff 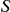.

The 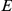-value associated with a peptide of score 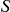 using the APP 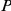-value will therefore be
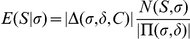
where the spectrum-specific 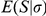 represents the 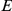-value for a hit with score 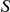 when the spectrum 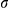 is used as the query and 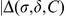 represents the total number of peptides in the set 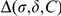. When cast in the aspect of per spectrum target-decoy approach, 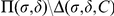 represents the largest possible decoy database, which is supposed to provide numerically the finest 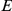-values for candidate peptides in the target database. (The symbol 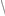 is called “setminus”. 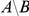 can be called 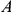 minus 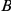 in the set sense or called complement of 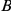 provided that set 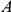 is the largest set considered and every set is a subset of 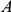.) Let 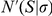 be the (weighted) number of peptide hits in the target database with score greater than 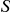. The per spectrum target-decoy approach will have
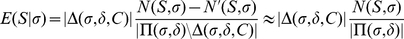
where the last result comes from 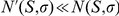 and 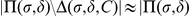 for any practical applications.

For a typical molecular mass of 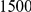 Dalton (Da) and in the absence of weighting, 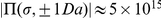. For a typical organismal database, such as that of *Homo sapiens*, the total number of peptides within the molecular mass range without any condition is only 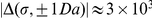. Therefore, 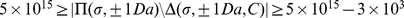, and 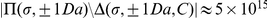. In the presence of peptide weighting, one still has 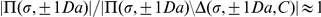. Therefore, 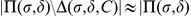. As for 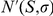 versus 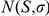, by definition 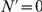 for best target hit and 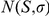 typically increases much faster than 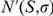 when 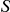 is lowered, thus 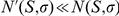, a fact also observed in reference [24]. Consequently, 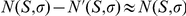 is a very good approximation. Therefore, the APP statistics also serve as the best per spectrum target-decoy statistics. The only question now is how does one get the score distribution of APP?

It turns out that if the score of a peptide is the sum of *local* contributions, meaning each term in the sum is uniquely determined by specifying a fragment's m/z value, then it is possible to construct the score histogram of APP via dynamic programming [10], [24]. When there exists intrinsically nonlocal contribution in peptide scoring, it is no longer possible to obtain the full histogram by dynamic programming. However, it is still possible to estimate the *de novo* rank via a scaling approach [15] similar to that used in statistical physics. The key point, as will be shown later, is that for the four scoring functions implemented in RAId_aPS, by using the APP statistics, it is no longer critical to theoretically characterize the score distribution obtained from the database search. This is because the 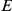-value obtained via RAId_aPS does agree well with the textbook definition. The APP statistics employed by RAId_aPS may be extended to provide robust spectrum-specific statistics for scoring functions that do not have theoretically characterized score distributions. One advantage to having a method that can provide robust spectrum-specific statistics for different scoring functions is that if the 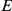-value reported by each method agrees with its definition, one can *compare* and *combine* search results from different search methods [9].

## Methods

### Basic Dynamic Programming Algorithm

To generate the score histogram of APP in a speedy manner, RAId_aPS does not score every possible peptide individually. As a matter of fact, it is impossible to score every possible peptide individually. For example, consider a typical parent ion molecular mass of 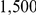 Da. It can be shown that the TNPP within 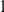 Da of this molecular mass is more than 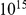. Even if one has a simple scoring function and a fast computer that can score one hundred millions peptides per second, it will take more than 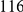 days of computer time to generate the score histogram for a single spectrum.

In real application, one needs to analyze a spectrum in a short time. How could one achieve this? One may use a 1-dimensional (1D) mass grid to encode/score APP [10], [24]. At each mass index of the grid, the local score contribution associated with all partial peptides reaching that location is computed only once and this information may be propagated forward to other mass entries via dynamic programming, making it possible to generate the score histogram of APP without individually scoring all peptides. In the score histogram, instead of counting number of peptides associated with a certain score, it is also possible to weight each peptide sequence according to its elemental composition. For a peptide sequence 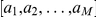, one may assign it a weight [10], [24] 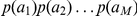 with 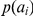 being the emitting probability of amino acid 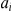.

**Figure 1:**
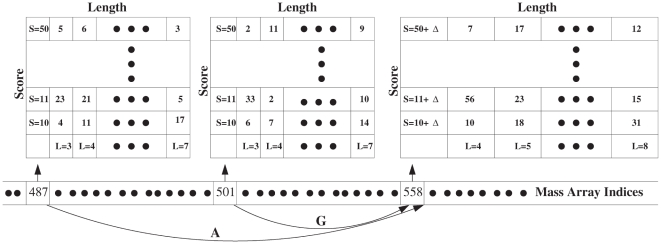
Illustration of APP mass grid with internal structure. In addition to show the basic mass grid, this figure illustrates,using the peptide lengths as an example, the possibility of including additional structures in the (raw) score histogram associated with each mass index. The basic idea of obtaining the score histogram via dynamic programming is explained in the Method section. The key step to incorporate additional structure is to let the (weighted) count associated with each (raw) score be further categorized by the lengths of partial peptides reaching each mass index. In the end, one will apply the length correction factor to the raw score to obtain the real score histogram. Apparently, one may also keep track of the number of 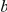 (
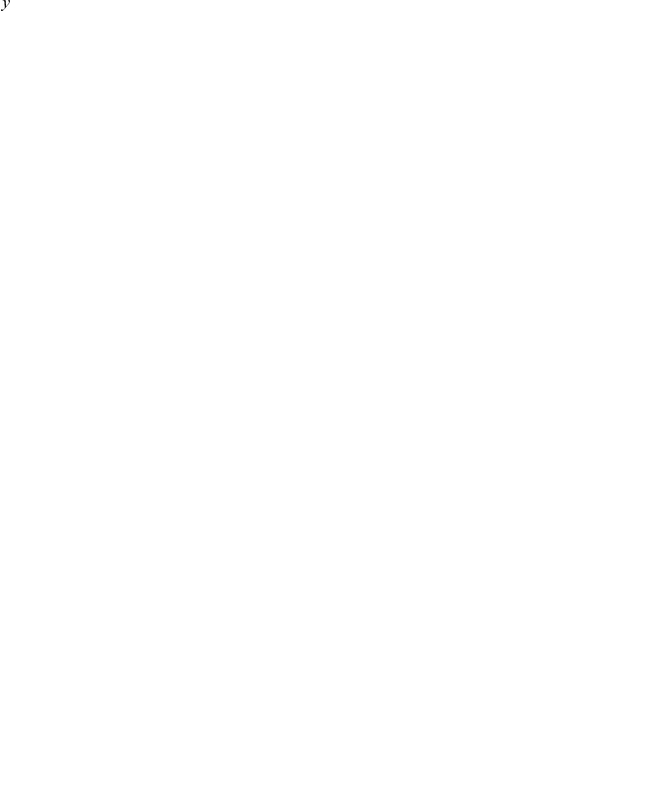
) peaks accumulated within the raw score histogram. Again, the factorial contribution can be added at the end prior to the construction of the final score histogram.

For illustration purposes, the mass grid of 1Da resolution is used in Figure 1. Each mass index contains a score histogram, with each entry in the left column indicating a score and the corresponding entry at the right column recording the number of partial peptides reaching that mass index with that score. The score histogram is obtained using a backtracking update rule. For example, at the mass grid 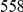, the local score contribution from evidence peaks in the spectrum is assumed to contribute 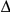 amount of score. Looking back to mass grid 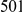 (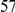 Da less than 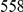 Da), one knows that by attaching a glycine residue to the partial peptides reaching mass index 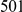 one will then advance these peptides to index 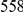. Similarly, any partial peptides reaching mass index 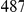 will move to mass index 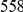 by adding an alanine residue. Therefore, at mass index 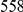 the score histogram is the superposition of score histograms associated with the other twenty lighter mass grids corresponding respectively to the twenty amino acids. For simplicity, the illustration is drawn as if there are only two amino acids, glycine and alanine. When one weights each peptide by its elemental composition, the counts next to the scores in the histogram are weighted and no longer integers. For example, the weighted count 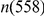 at mass index 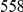 will be given by 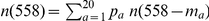 where 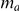 is the mass of amino acid 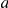 rounded to the nearest Da and 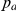 is the emitting probability associated with amino acid 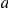. In addition to attaching a score histogram to each mass grid, one may also include other internal structures such as peptide lengths, peak counts, etc. as shown in the caption of Figure 1. When one suppresses the score and only counts number of partial peptides reaching a certain mass index, the update rule readily provides the total number of peptides within a given mass range.

### Spectral Filtering

Before describing the scoring functions, the major component of peptide database search tools, we first mention spectral filtering, an often under-emphasized but equally important ingredient. Starting with a raw tandem mass spectrum, spectral filtering produces a processed spectrum that is used to score candidate peptides in the database. Apparently, information kept in the processed spectrum plays an important role in the effectiveness of a tool's performance in database searches. Customized for different scoring functions, different filtering strategies are employed by different search tools. In order for RAId_aPS to capture the essence of a scoring function, it is very important for RAId_aPS to produce, for every input raw spectrum, a filtered spectrum that is as close as possible to the one produced by other search tool's filtering protocol. For most search tools, the filtering heuristics are not clearly documented. For that reason, it becomes necessary to delve into the source code of the search program to find out each method's spectral filtering protocol. We are thus limited to search tools whose source programs are available or those with filtering strategies clearly documented.

For RAId score, the spectral filtering strategy was described in an earlier publication [11]. For Hyperscore [25], XCorr [17], and K-score [26], [27], the details of spectral filtering will be described in Text S1. Since the SEQUEST source code is not available, for XCorr score we attempt to replicate the filtering of Crux [20], a search method that has been shown to reproduce SEQUEST XCorr [20]. That the filtering strategies extracted are accurate can be seen from Figure S1. The spectral correlation histograms between the filtered spectra produced by RAId_aPS's Hyperscore/XCorr/K-score with the filtered spectra from X!Tandem/Crux/X!Tandem(with K-score plug-in) show that RAId_aPS is able to produce filtered spectra identical to those generated by the canonical programs. Although the spectral filtering strategies associated with various search tools investigated seem stable, it is still possible that the developers may change their filtering strategies in the future. When that happens, one should be able to update RAId_aPS to reflect the filtering changes provided that the source programs are still accessible and clearly documented.

**Figure 2:**
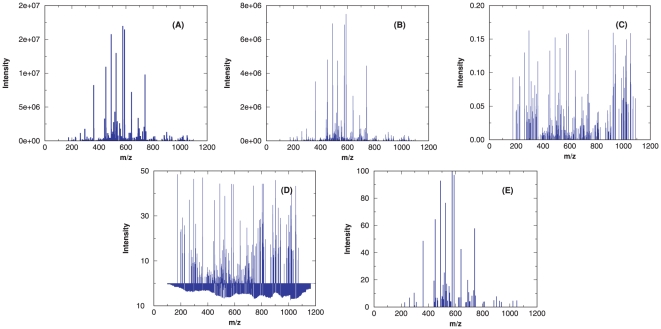
Example processed spectra from different scoring functions versus the original spectrum. The centroid spectrum used has a parent ion mass of 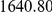 Da. In panel (A), the original spectrum is displayed; (B) shows the processed spectrum generated by the filtering protocol of RAId_DbS scoring function; (C) exhibits the processed spectrum generated by the filtering protocol of K-score; while (D) and (E) correspond respectively to the processed spectra produced by XCorr and Hyperscore.

**Figure 3:**
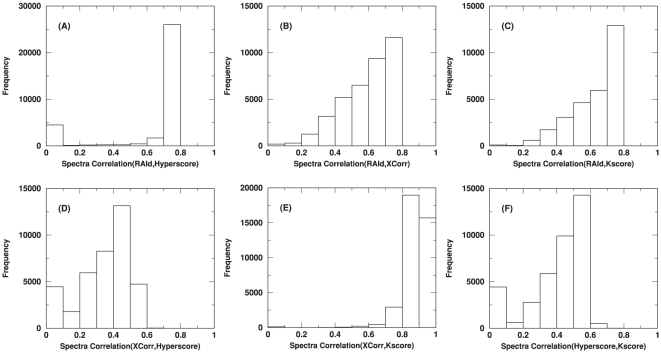
Histograms of correlations between filtering strategies. Used in this plot are 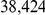 raw centroid spectra from the ISB data set [28]. Each raw spectrum will have four different processed spectra come from each of the four different filtering strategies. The mass fragments of every filtered spectrum are then read to a mass grid. The spectrum is then viewed as a vector with non-vanishing components only at the populated component/mass indices. One then normalizes each *filtered* spectrum vector to unit length. An inner product of any two filtered spectral vectors represents the correlation between them. When the spectral quality does not pass a method-dependent threshold, the corresponding filtering protocol may turn the raw spectrum into a null spectrum without further searching the database. For a given pair of filtering methods and a raw spectrum, if each of the two filtering methods produces a nonempty filtered spectrum, one may turn those filtered spectra into spectral vectors and compute their inner product, i.e., their correlation. For each pair of filtering methods, these inner products are accumulated and plotted as a correlation histogram. All six pairwise combinations are shown.

Instead of elaborating on various filtering strategies, let us first use a experimentally obtained spectrum to demonstrate the effect of spectral filtering employed by different methods. Figure 2 shows the raw spectrum, and the filtered spectra processed by the four scoring methods mentioned. The general trend is as follows: RAId score usually produces the filtered spectrum that resembles the original spectrum the most; Hyperscore filtering also produces a processed spectrum that is similar to the original spectrum; for XCorr and K-score the filtered spectra in general look quite different from the original spectrum. The differences in the filtered spectra might be a major factor contributing to the fact that different search methods have different and often complementary strengths. The correlation between any pair of filtering strategies can be quantified. Starting with a large set of raw spectra, one may process these spectra with a pair of different methods. For each raw spectrum, one obtains two different filtered spectra and can compute their correlation. The correlation between every pair of filtered spectra can then be collected to form the correlation histogram, reflecting the correlation between a pair of filtering strategies. Figure 3 and Figure S2 exhibit the correlation histograms between each pair of filtering strategies using different data types: centroid (A1–A4 of ISB data set [28], Figure 3) and profile (NHLBI data set [4], Figure S2). The large correlation between XCorr and K-score may be the cause of their significant scoring correlation observed.

### Scoring Functions

To better express the scoring functions, let us first define the following notations. For a given peptide 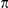, the set of corresponding theoretical mass over charge (m/z) ratios taken into consideration by a scoring function is called 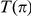, which is also used to indicate the number of elements in the set 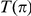 whenever no confusion arises. The set 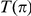 varies from software to software. However, the fragmentation series 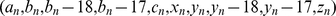 include what most methods consider. The Heaviside step function 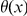 is defined by 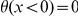 and 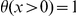. We introduce 
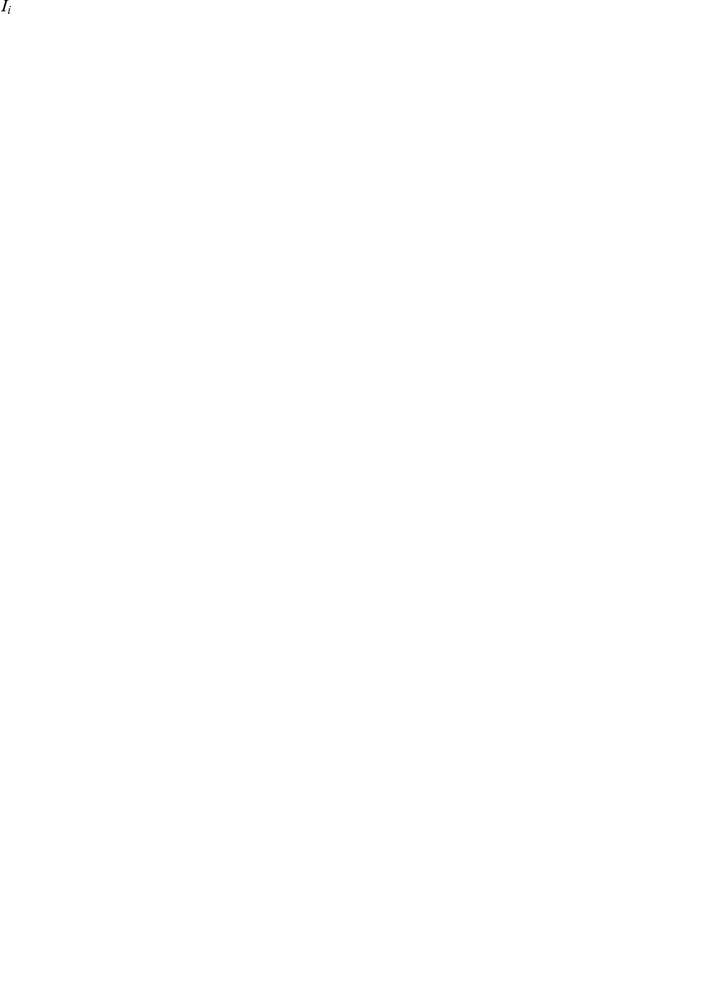
 as a shorthand notation for 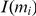, the peak intensity associated with theoretical mass 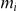 in the *processed* spectrum. In an experimental spectrum, the mass giving rise to 
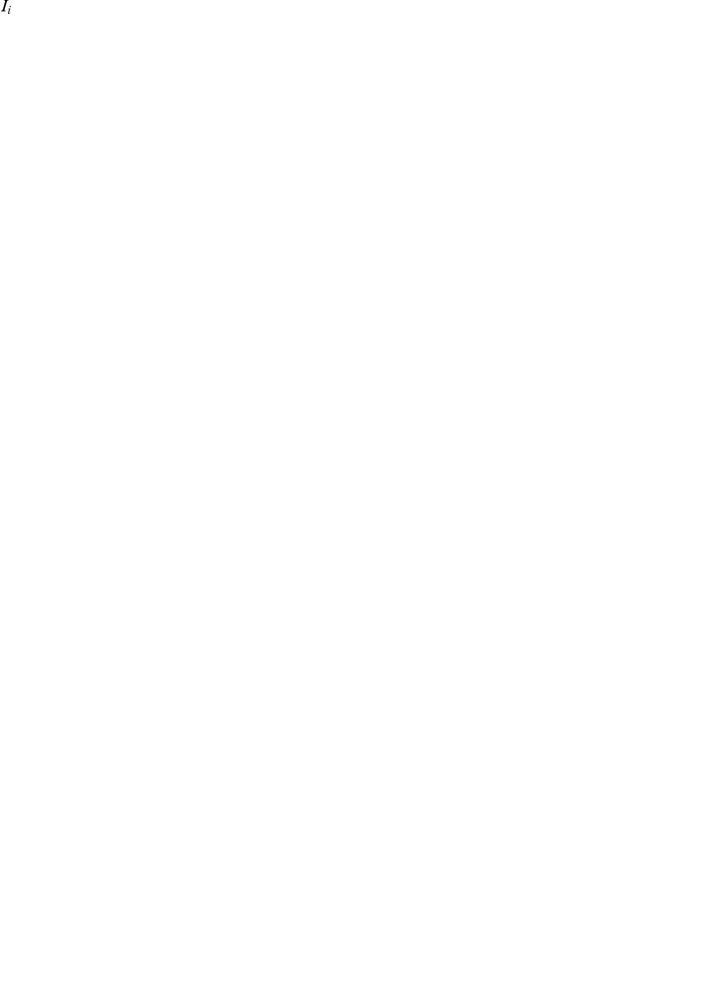
 usually does not coincide with 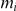. The absolute difference between the experimental mass (giving rise to 
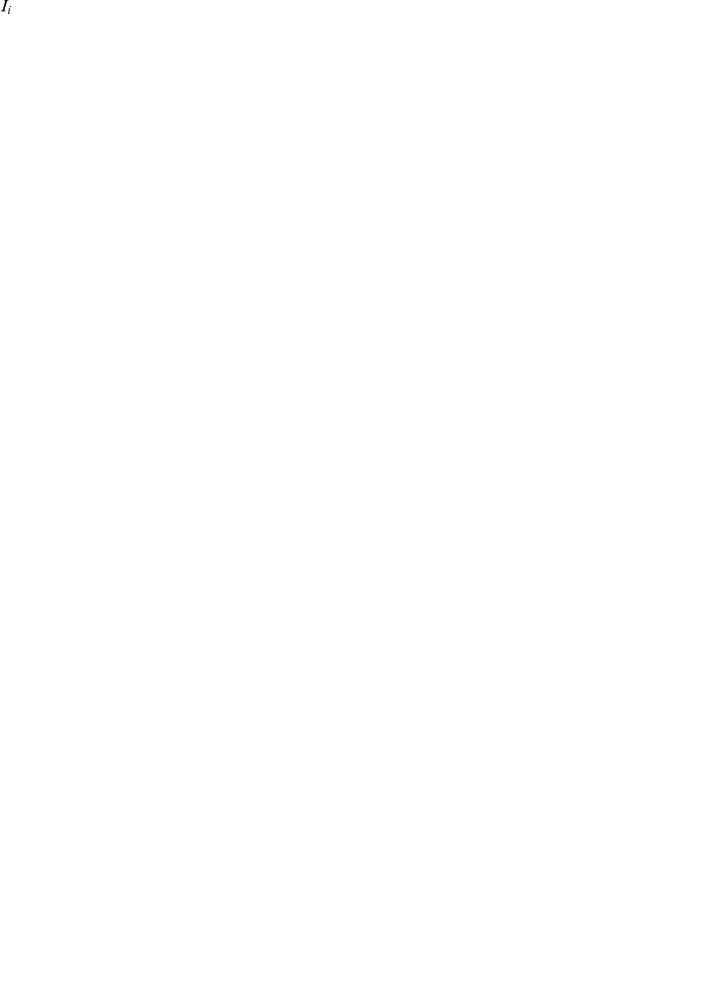
) and the theoretical mass 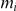 is denoted by 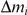. The notation 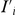 is used in place of 
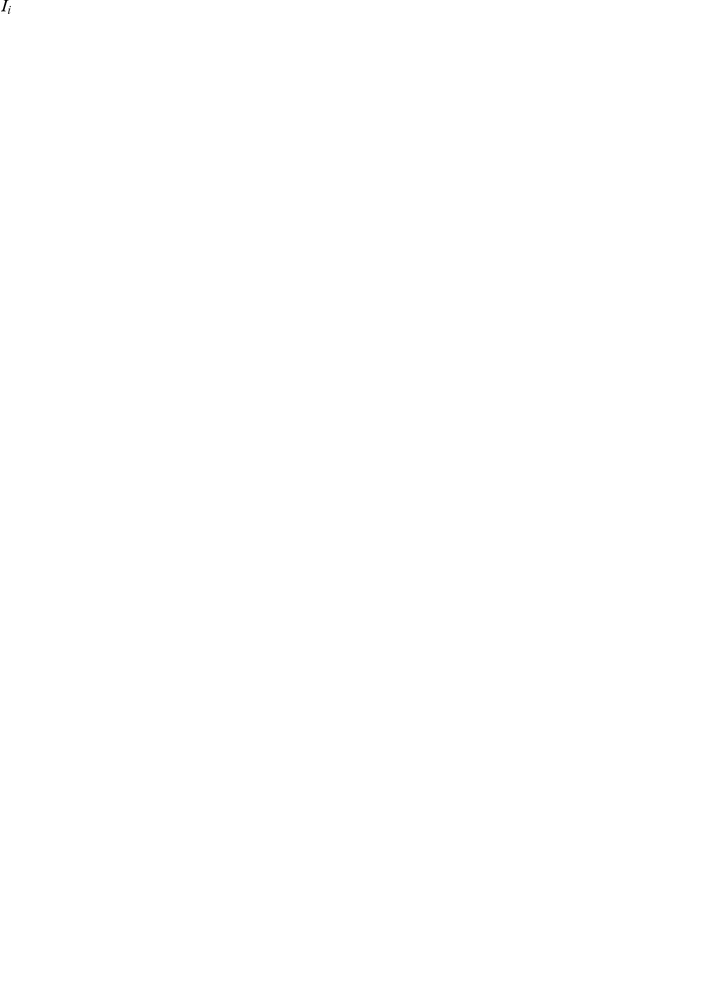
 when the preprocessing of the spectrum involves a nonlinear transformation of the peak intensity or involves generation of additional peaks. We now list the four different scoring function implemented:
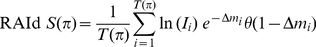
(1)

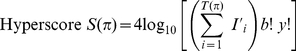
(2)

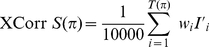
(3)

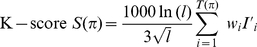
(4)


The first scoring function listed is employed by RAId_DbS [11]; the second one mimicks the Hyperscore (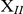) of X!Tandem [13]; the third one mimicks the XCorr score used in SEQUEST and is similar to what was implemented in Crux [19], [20]; the last one mimicks K-score [26], a plug-in for X!Tandem. For the RAId score, the set 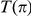 includes only the 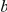
- and 
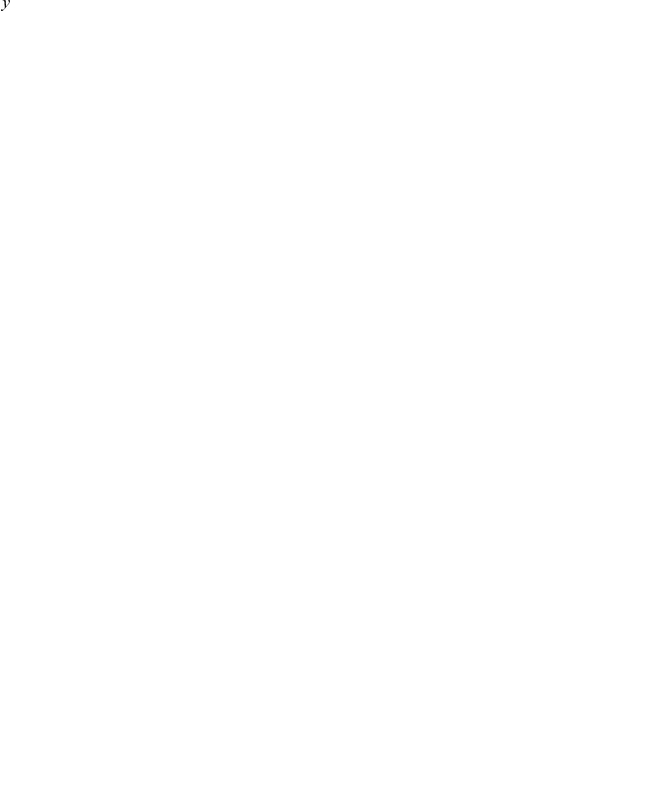
-series peaks. For the Hyperscore, 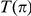 includes 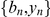. For XCorr, 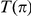 includes 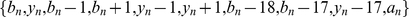 with the corresponding weights given by 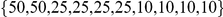. For K-score, 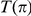 includes 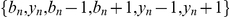 with the corresponding weights given by 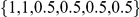. To speed up the code, we have chosen to rescale the weights for XCorr (see the “Crux Filtering and XCorr” section of Text S1 for detail).

Very often it is useful to include the peptide length in the scoring of a peptide. Using RAId score as a simple example, two peptides of length 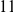 and 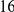 may achieve the same raw score 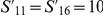, sum of the logarithm of evidence peak intensity. A longer peptide consists of a longer list of theoretical peaks to look for and may thus score higher by chance. RAId_DbS scoring function [11] deals with this issue by dividing the raw score by the length of the theoretical peak list. Upon doing so, one has 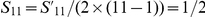 and 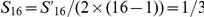. This score normalization may help in discriminating true positives from false positives. The other scoring function utilizing the peptide length information is the K-score. Hyperscore, employed by X!Tandem, uses a slightly different score renormalization strategy. Inside the logarithm, the Hyperscore contains two factorials, 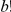 and 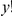. For each candidate peptide, 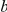 (
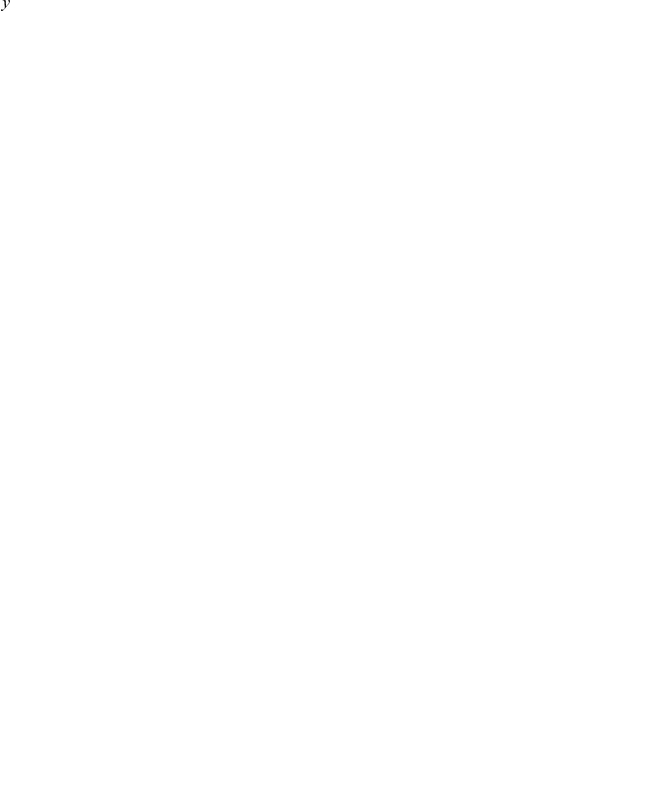
) represents the total number of 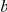-series (
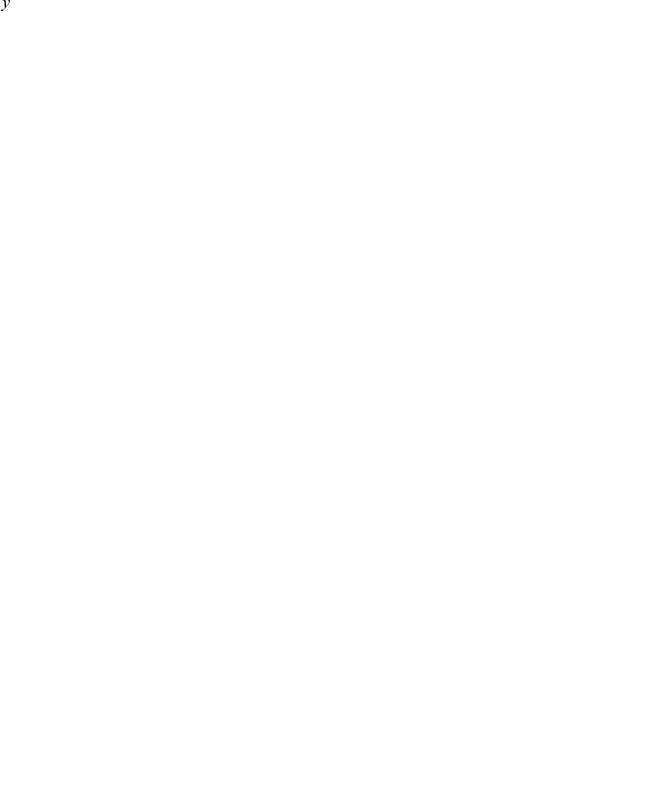
-series) evidence peaks found in the spectrum. At any specified mass index in the mass grid, unlike the peak intensity associated with that index, neither the peptide length nor the total number of the b (y) peaks has a unique corresponding value. Therefore, one needs to extend the basic algorithm outlined in the previous subsection to accommodate these additional information needed for scoring.

As documented in reference [10], it is possible to introduce additional structures in the score histogram associated with each mass index. The flexibility to introduce additional structures of various dimensions makes RAId_aPS a versatile tool: it can accommodate the scoring functions that utilize length information or the number of 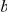-series (
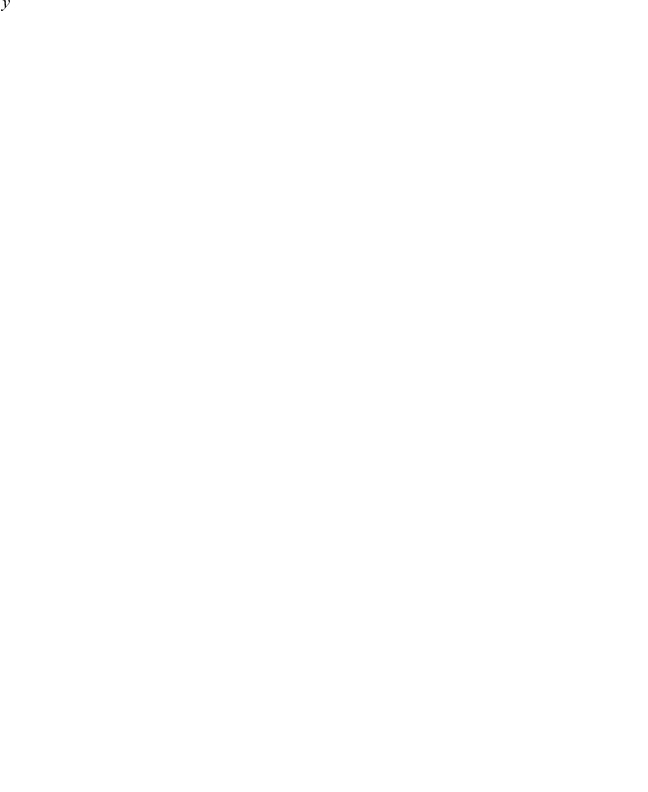
-series) peaks to compute the final peptide score. Using peptide length as an example, Figure 1 demonstrates the inclusion of additional structures. More detailed exposition about the inclusion of internal structures can be found in reference [10].

**Figure 4:**
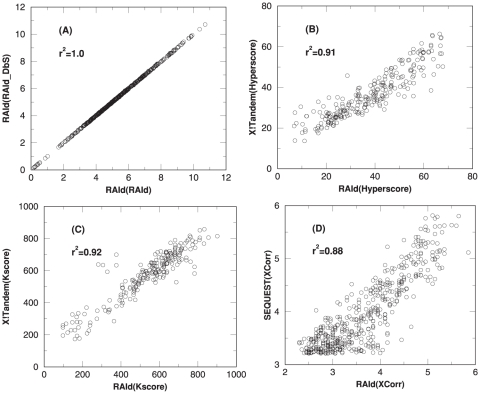
Score correlations. A subset of the ISB centroid data set [28] was used to perform this evaluation. For each scoring function, when the best hit per spectrum (analyzed using the analysis program that the scoring function was originally used for) is a true positive, that candidate peptide is scored again using the corresponding scoring function implemented in RAId_aPS. Each true positive best hit thus gives rise to two scores and plotted using the following rule: the first score is used as the ordinate while the second score (from RAId_aPS) is used as the abscissa. Including 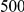 spectra, panel A is for the RAId score. Panel B is for Hyperscore and contains 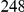 spectra. The result of K-score is shown in panel C with 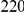 spectra. Shown with 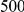 spectra, panel D documents the results for XCorr.

Although the spectral filtering parts of various scoring functions are replicated exactly, a candidate peptide may receive different scores from RAId_aPS and the original programs. This phenomenon can be seen in Figure 4: the ordinate of each data point displays the search score of the best hit of a centroid spectrum using the original programs, while the abscissa of the same data point shows the score reported by RAId_aPS. The corresponding plots for profile data are shown in Figure S3.

The major source of score difference is due to RAId_aPS's omission of *heuristics* while implementing a published scoring function. For each scoring function, many scoring heuristics are present in the source code. While some of the heuristics cannot be included via dynamic programming, all these heuristics are either not described or not justified in the original papers. For these reasons, RAId_aPS does not include those unpublished heuristics. Therefore, the Hyperscore/XCorr/K-score scoring functions implemented in RAId_aPS should be regarded as our attempt to mimick the original Hyperscore/XCorr/K-score scoring functions. Although the scoring functions we implemented are not exact replicas of the original ones, due to omission of heuristics, we can see from Figure 4 (and also Figure S3 when tested on profile data) that there exist strong correlation between each scoring function implemented in RAId_aPS and the original, corresponding scoring function. In other words, the scoring functions implemented in RAId_aPS do capture the essence of these original scoring functions.

### APP Statistics: practical implementation

In the APP statistics section, we described how to use APP statistics to obtain 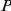-values and 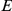-values with or without weighting each peptide by its elemental composition. In this subsection, we will complement the theoretical presentation by describing some pragmatic aspects of the implementation.

In order to build the score histogram quickly, it is necessary to discretize the score, thereby compromising to some degree the score precision. However, this rounding of scores does not affect peptide scoring when using RAId_aPS as a database search tool or a tool to provide statistical significance for a list of peptides. Specifically, the evidence score collected at each mass index is stored in two formats: one with much higher precision and the other rounded to nearest integer. The rounded values are used in dynamic programming to propagate the score histogram forward, facilitating a speedy construction of the score histogram. The slight error introduced in individual peptide scoring does not influence the accuracy of the score histogram much since these errors largely cancel each other when lumping the scores into a histogram. In the database search mode, RAId_aPS will sum the high precision evidence scores in the mass indices traversed by the candidate peptide being scored. Therefore the score associated with each candidate peptide in the database search mode has a better resolution than that in the score histogram. To obtain the statistical significance associated with each candidate peptide, RAId_aPS performs an interpolation procedure to obtain the 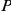-value,
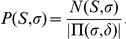
Multiplying the 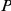-value by the number of qualified peptides 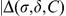 in the target database provides the 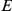-value
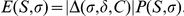



### APP Statistics including PTM amino acids

Since proteins do contain PTM amino acids, it is important for peptide identification tools to consider amino acid modifications in the statistical analysis. By scoring only qualified peptides, database search methods have little problem including PTM amino acids provided that the score distribution is theoretically characterizable. For APP based statistics, even though the score distribution is not always characterizable, information from *qualified* peptides in database search may be used to generate the emission probabilities of all the amino acids, PTMs included, needed for APP based statistics.

Given a parent ion mass and a database, once the allowable PTMs are specified, the number of peptides along with possible types of modifications are fixed. This renders a parent-ion-mass specific and database specific emission probabilities for PTMs. Nevertheless, the list of qualified peptides may vary with molecular mass error tolerance while the allowable PTMs may also vary with users' specification for a search. Once the list of qualified peptides for a spectrum is given, the emission probabilities of each amino acid (including PTMs) are computed as follows: for each amino acid 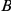, RAId_aPS first counts the number of occurrences of the unmodified amino acids 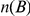 and the number of occurrences 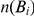 of 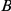 modified into a different form 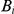, with 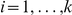. RAId_aPS then proportionally distributes the emission probability 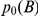 associated with amino acid 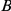 to all the possible modified forms using the following formulas
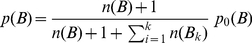
(5)

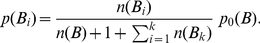
(6)Effectively, one pseudocount is always given to each unmodified amino acid.

Therefore, for a given list of peptides, RAId_aPS will count the total number of distinct amino acids modifications. In principle, RAId_aPS can incorporate all those modified amino acids in the score histogram construction. However, for reasons to be described below, RAId_aPS retains no more than the ten most abundant PTMs in calculating the new emission probabilities. First, the estimated emission probabilities of PTMs become less trustworthy when the occurrences of those PTMs are rare. Second, inclusion of many PTMs can slow down the process, although not very much. Assume that one incorporates 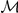 modified amino acids in the score histogram construction, the number of trace backs per mass index becomes 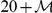 instead of 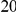. This introduces a factor of 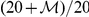 compared to the original construction. Further, the size of score array associated with each mass index needs to be larger than before and thus require more time to compound the score histogram. This approximately introduce another factor of 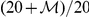 to the computation speed. Thus, introducing 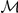 modifications will introduce a multiplicative factor of 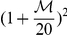 to the computation time. To ensure that the average run time does not grow more than two fold, we set the maximum 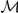 allowed to be ten. The new set of *normalized* background frequencies (with the most abundant PTMs included) may then be fed into RAId_aPS to compute the corresponding APP score histogram. The histogram obtained is then used to calculate the statistical significance of each reported peptide.

Although rare PTMs in the peptide list might be omitted in constructing the APP score histogram, the impact on the statistical significance accuracy is minute. For if one were to include those PTMs, due to their small normalized emission probabilities, peptides containing those PTMs would be weighted substantially less than others and thus would not significantly affect the shape of the score histogram. As for the emission probability 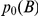 —needed in eqs. (5–6)— associated with amino acid 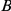, one may use either known amino acid background frequencies such as the Robinson-Robinson [29] frequencies or can calculate the number of occurrences of all amino acids in a *parent-ion-mass-specific* and *database-specific* manner. The former approach is adopted by RAId_aPS when the number of peptides (provided by the user or extracted from the database) is less than 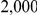; otherwise, the latter approach is employed. There exists, of course, room for improvement in terms of including PTMs in the APP statistics. Alternatives are currently under investigations.

### Combining Search Results from Different Scoring Functions

When the user select multiple scoring functions in mode (iii) and mode (iv), RAId_aPS is able to combine statistical significances reported by the different scoring functions. For database search (mode (iv)), the protocol to combine search results is identical to what was described before [9]. In this section, we will briefly review this method.

For a given spectrum 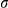, to combine search results from 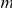 scoring functions (say scoring function 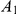, 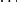, 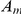), we first construct a union peptide list 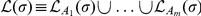, where 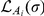 is the reported list of peptide hits by method 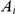 for spectrum 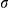. A peptide in the union list has at least one, and may have up to 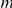 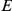-values derived from APP 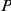-values, depending on how many scoring functions reported that specific peptide in their candidate lists. Each of the 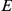-values associated with a peptide will be first transformed into a *database* 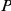 *-value* [9], representing the probability of seeing at least one hit in a given random database with quality score larger than or equal to 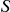. If one assumes that the occurrence of a high-scoring random hit is a rare event and thus can be modeled by a Poisson process with expected number of occurrence 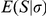, one may obtain the database 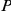-value mentioned earlier via
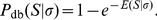
(7)


The database 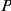-value of peptide 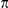 is set to one for methods that do not report 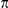 as a candidate. After this procedure, each peptide in the list 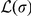 has 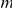 database 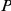-values 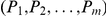. Assume that these 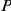-values are independent, the combined 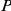-value (with 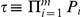) for peptide 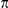 is given by [9]

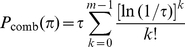
(8)Once 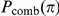 is obtained, we may invert the formula in Eq. (7) to get a combined 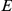-value 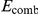 via
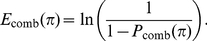
(9)We then use 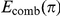 as the final 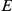-value to determine the statistical significance of peptide candidate 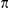, similar to what is used in reference [30]. From a theoretical stand point, one might ask whether or not eq. (8) always gives rise to a smaller combined 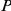-value than any of the input 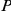-values. The answer is no. For example, consider 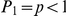 and 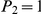. One then has combined 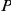-value 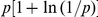 larger than 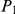. Readers interested in more details are referred to Appendix B of reference [9].

The combining 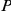-value strategy outlined by eqs. (7–9) is founded on the assumption that 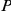-values resulting from different search scores are independent. That is, the resulting significance assignment is valid only when scoring functions considered are uncorrelated, or at most weakly correlated. In our earlier investigation [9], we found that although many scoring functions are looking for similar scoring evidences, the pairwise correlations among scoring functions investigated are weak, perhaps due to different spectral filtering methods employed. The weak pairwise correlations among different scoring functions implies that the outlined strategy above may still provide decent significance assignment. How to *properly* take into account method correlations while combining the search results is of course a very important and open problem.

Suppose one has obtained a list of candidate peptides from some analysis tools that provides only crude statistical significance assignment or no significance assignment at all, it is possible to upload this list of peptides along with the spectrum to RAId_aPS to get a reassignment of statistical significance via mode (iii) of RAId_aPS. The fundamental idea here is to first obtain the score histograms corresponding to the list of scoring functions selected. With the histograms constructed, one can generate the 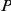-values for any score specified. Therefore, for a chosen scoring function and a given list of peptides, RAId_aPS can provide for each peptide an APP 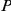-value by scoring each peptide and then inferring from the normalized score histogram.

In practical implementation, RAId_aPS sorts the list of peptides according to their molecular masses and identifies their corresponding mass indices on the mass grid. Using these indices as terminating points, but one at a time, RAId_aPS constructs score histograms assuming that the parent ion weight is given by the mass indices considered. Each peptide in the list is then rescored using the user-selected scoring versions implemented in RAId_aPS and the 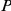-values corresponding to these scoring functions are obtained. If no further information other than a flat list of peptides is given, RAId_aPS will combine these 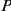-values using eq. (8) and return a combined 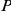-value for each peptide in the list. When the number of qualified database peptides is known –which is the case if one directly uploads to RAId_aPS any of the output files of Mascot, SEQUEST, or X!Tandem– RAId_aPS will first transform the 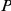-values into 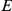-values and then into database 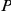-values (eq. (7)). For each peptide in the list, RAId_aPS will then combine their database 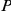-values using eq. (8) and then obtain the final 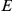-value via eq. (9).

## Results

### 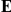-value Accuracy

In the APP statistics subsection of Technical Background, it was demonstrated that statistical significance assignment based on the APP score histogram is *spectrum-specific*. However, one must verify 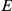-value accuracy before claiming that accurate spectrum-specific statistics are achieved via APP statistics. A straightforward way to test 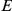-value accuracy [11] is to compare the averaged number of false positives (the textbook definition) versus reported 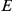-value using a spectral dataset resulting from a known mixture. To be specific, one will first eliminate true positives from a database, and then use the spectra from a known mixture as queries to look for peptide hits. Since the true positives are removed from the database beforehand, all the peptide hits are false positives. One then aggregates all the false positives together –there might be many false positives from one spectrum– and then sorts them in ascending order of 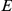-value. Let 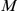 be the total number of spectra used for evaluation and let 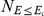 be the total number of false positives with 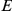-values smaller than or equal to 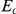. If the 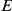-values reported are accurate, one expects to see that
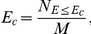
subject to fluctuations due to finite sampling.

**Figure 5:**
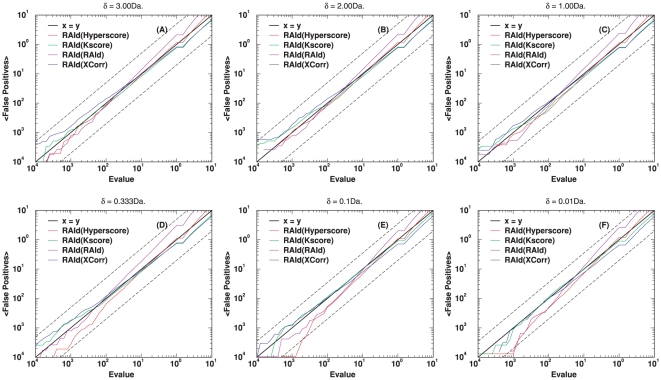
E-value accuracy assessment. The agreement between the reported 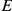-value and the textbook definition is examined using centroid data (A1–A4 subsets of ISB data set). The random database size used is 500 MB. The molecular weight range considered while searching the database is 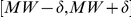. In each panel, the dashed lines, corresponding to 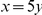 and 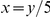, are used to provide a visual guide regarding how close/off the experimental curves are from the theoretical curve.

Figures 5 and S4 assess 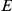-value accuracy when 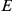-values are obtained from APP 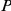-values. Figure 5 displays, based on searching a random database of size 500MB, the measured average number of false positives as a function of the reported 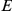-value. The six-panel figure demonstrates statistical stability against allowed mass error. For parent ion mass of 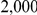 Da, what is displayed in Figure 5 covers the resolution range from 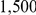 ppm to 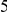 ppm. Figure S5 displays the corresponding result for profile data. The statistical stability shown is important since the use of high resolution mass analyzers such as Orbitraps have gained popularity. Figure S4, using the NCBI's nr database, examines the 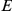-value accuracy when used in biological context. Since the biological database is not a collection of random peptides, the validity of statistical theory founded on random databases should be tested. As shown in Figure S5, the same statistical robustness holds for both centroid and profile spectra while searching the biological protein database tested.

Both the centroid data set and profile data set are tryptic and are identical to the ones used in reference [4]. The 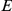-value for a peptide hit is obtained by multiplying that peptide hit's APP 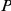-value by a numerical factor 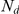, the number of qualified database peptides with similar masses. In terms of enumerating qualified peptides, we employ the RAId_DbS strategy. Specifically, we further divide the qualified peptides into ones with correct and incorrect N-terminal cleavages [11] and have separate counters for them. If a candidate peptide has correct N-terminal cleavage, its 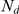 factor is the total number of database peptides with both correct N-terminal cleavages and with masses similar to that of the peptide considered; otherwise, it will have a considerably larger 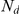 factor that counts *all* database peptides with masses similar to that of the peptide considered. The protein database used is the NCBI's nr (same version as in reference [11]) with identical cluster removal procedure [11]. As shown in Figure 5 and Figures S4, S5, the 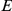-values reported by RAId_aPS using the various scoring functions implemented are within a factor of five of the textbook definition. For any two scoring functions, if they are independent, one may combine the statistics using eqs. (7–9) and the combined 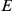-value should also follow the theoretical curves.

How well the combined 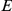-values reported trace the theoretical line can be used as a measure of how independent these two scoring functions are, provided that each scoring function already has 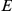-value reported in agreement with the textbook definition. As in reference [9], the combined 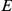-value from any two methods in general shows a larger deviation from the textbook definition. This may be due to correlations between search methods. We are currently investigating the possibility of taking into account the search method correlation, which we suppose to be spectrum-specific too, while combining the statistics. We will incorporate the corrected statistics into RAId_aPS if the investigation along this direction turns out to be fruitful.

### Combine Database Search Results

The primary feature of RAId_aPS is the ability to combine, in a statistically sound way, search results from different scoring functions. If the retrieval performance of each scoring function implemented is poor, then even if one combines the search results, the final outcome might still be poor. Below we assess the retrieval performance of each scoring function implemented using the Receiver Operating Characteristic (ROC) curves.

### First assessment of scoring functions

**Figure 6:**
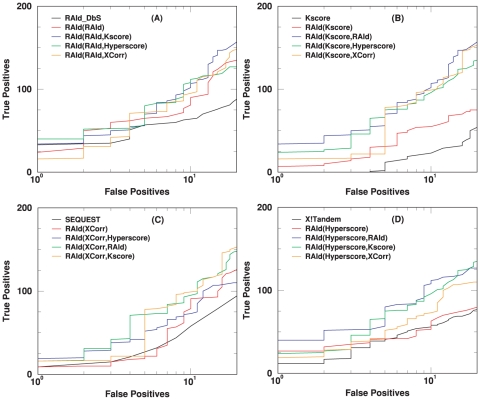
ROC curves for the centroid data (A1–A4 of the ISB data set [28]). For each of the four scoring functions considered, a set of ROC curves is shown. These ROC curves include the results from running the designated program associated with that scoring function, the results from running RAId_aPS in the database search mode, and the results from combining with each of the three other scoring functions. Panel (A) shows the results from RAId score, whose designated program is RAId_DbS. Panel (B) displays the results from K-score, whose designated program is X!Tandem. Panel (C) exhibits the results from XCorr, which is mostly employed by SEQUEST. Panel (D) presents the results from Hyperscore, whose designated program is also X!Tandem. Instead of using only XCorr (like RAId_aPS), SEQUEST first selects the top 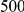 candidates using SP score. As shown in panel (C), for centroid data there is an advantage to filtering candidates with the SP score. However, it is also seen that by combining XCorr with either RAId score or Hyperscore, equally good results can be attained without introducing the SP score heuristics.

**Figure 7:**
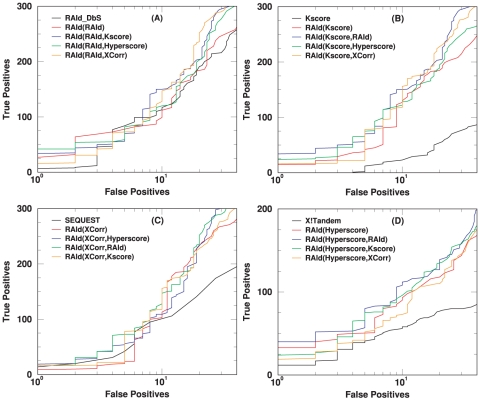
ROC curves for the centroid data (A1–A4 of the ISB data set [28]) when considering only the best hit per spectrum. For each of the four scoring functions considered, a set of ROC curves is shown. These ROC curves include in the consideration only the best hit per spectrum from running the designated program associated with that scoring function, the best hit per spectrum from running RAId_aPS in the database search mode, and the best hit per spectrum from combining with each of the three other scoring functions. Panel (A) shows the results from RAId score, whose designated program is RAId_DbS. Panel (B) displays the results from K-score, whose designated program is X!Tandem. Panel (C) exhibits the results from XCorr, which is mostly employed by SEQUEST. Panel (D) presents the results from Hyperscore, whose designated program is also X!Tandem. Instead of using only XCorr (like RAId_aPS), SEQUEST first selects the top 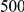 candidates using SP score. As shown in panel (C), for centroid data there is advantage to filter candidates with the SP score. However, it is also seen that by combining XCorr with either RAId score or Hyperscore, equally good results can be attained without introducing the SP score heuristics.

Here we investigate the performance of the four implemented scoring functions –RAId score, K-score, XCorr, and Hyperscore– each of which is a standard scoring function, often employed with program-specific heuristics, for a known search program. The retrieval efficiency is assessed using a centroid data set (Figure 6, ISB data set). Since many search methods report only one or very few candidate peptides per spectrum, we also include this type of ROC curve (Figure 7) where only the best hit per spectrum is taken from the search results. The performance of this *ad hoc* truncation apparently leads to better retrieval at small number of false positives, indicating the existence of false hits whose evidence peaks are homologous to that of the true positive(s) associated with a spectrum. We are currently investigating the impact of the existence of these types of false positives on the statistical significance assignment. The results will be reported in a separate publication. The corresponding plots when using a profile data set (NHLBI data set) are shown respectively in Figure S6 (similar to Figure 6) and Figure S7 (similar to Figure 7).

### Different ROC analysis

When the true positive peptides are not known *a priori*, there exist various strategies in classifying hits into true or false positives when making a ROC plot. These strategies, unfortunately, will make a notable difference in retrieval assessment. For example, in a cell lysate experiment of a certain organism, it is customary to estimate the number of false positive hits by introducing a decoy database during the data analysis. The main idea there is to first sort the peptide hits according to their scores. Then for each decoy hit, one assumes that there is just one corresponding false hit in the target database. This strategy has been used extensively [24]. ROC analyses done this way generally count false positives, which are highly homologous to the target peptides, towards true positives. This has two effects: an overcount of true positives and a undercount of false positives. As a consequence, the ROC curves will appear more impressive. To mimick this situation, we used BLAST to find in the NCBI's nr database highly homologous proteins to the target proteins used in the experiment and include those proteins in our true positive set. This strategy produces ROC curves shown as the solid curves of Figure S8. When compared to Figure 6 and Figure S6, the ROC curves produced by this strategy seem much more impressive.

Not counting highly homologous proteins as false positives would probably be agreeable. However, counting those peptides/proteins as true positives could be exaggerating. Therefore one may use a slightly different strategy: removing from consideration proteins homologous to the target proteins, which is called the cluster removal strategy [11]. The dashed curves of Figure S9 are ROC curves obtained this way. This strategy also produces slightly more impressive ROC curves than in Figure 6 and Figure S6. Apparently, this indicates the highly homologous false positive hits are the ones that degrade the retrieval performance. Thus, it can be useful to remove those false positives from consideration. Keeping only the best hit per spectrum turns out to be one way to achieve this goal.

### Combining Multiple Scoring Functions

Since different scoring functions have different spectral filtering strategies, it is often advantageous to combine the search results from several scoring functions. RAId_aPS provides a simple user interface, allowing users to select several scoring functions at a time. A example output when several scoring functions are selected is shown in Table 1.

**Table 1:** An output example of the combined *E-value* from RAId_aPS.

E_comb	RAId	Hyperscore	XCorr	K-score	Peptide
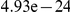	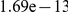	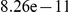	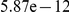	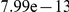	NYQEAKDAFLGSFLYEYSR
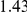	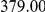	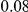	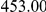	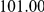	APTSAGPWEKPTVEEALESGSR
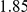	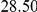	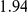	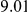	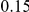	LERMTQALALQAGSLEDGGPSR
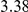	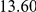	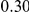	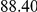	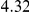	TEDQRPQLDPYQILGPTSSR
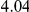	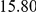	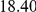	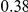	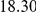	NYKAKQGGLRFAHLLDQVSR
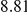	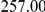	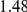	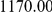	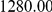	DTPMLLYLNTHTALEQMRR
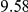	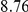	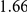	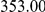	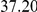	EKTESSGQETTAKCDRASKSR
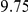	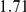	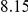	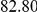	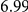	LLAQQSLNQQYLNHPPPVSR
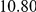	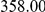	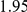	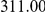	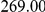	IQHGQCAYTFILPEHDGNCR

**Figure 8:**
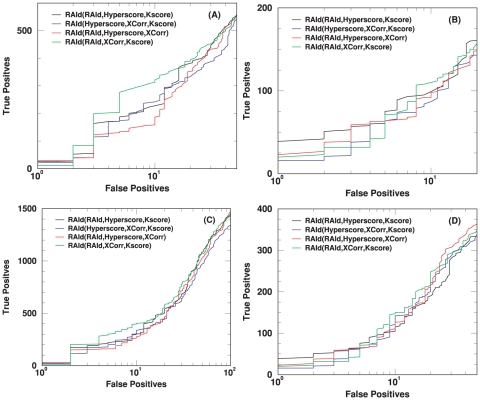
Illustration of RAId_aPS performance when combining three different scoring functions. Panel (A) shows the results from the profile data (NHLBI data set [4]), while panel (B) exhibits the results from the centroid data (A1–A4 of the ISB data set [28]). Panel (C) shows the results from the profile data but keeping only the best hit per spectrum, while panel (D) exhibits the results from the centroid data but keeping only the best hit per spectrum.

Figure 8 illustrates the performance when RAId_aPS combines three different scoring functions in its database search mode. Panels (A) and (B) of Figure 8 should be compared with Figure 6 and Figure S6 respectively. The ROC curves obtained by combining three randomly chosen scoring functions indicate better performance than individual scoring functions. Panels (C) and (D) should be compared with Figures 7 and Figure S7 respectively. The results in those plots are obtained from keeping only the best hit per spectrum prior to further analysis. As shown in those plots, the ROC curves obtained by combining three randomly chosen scoring functions indicate significantly better performance than individual scoring functions, except for the case of RAId_DbS.

### Other modes

Examples of using mode (iv) were already shown above. We demonstrate here other features of RAId_aPS to illustrate its versatility.

### Compute TNPP: mode (i)

Given a parent ion mass, RAId_aPS is also able to compute efficiently the TNPP associated with that molecular mass within a user-specified mass error. The user interface for computing TNPP is self-explanatory. One simply types in the molecular mass of interest, chooses a specific digesting enzyme or considers no enzymatic restriction by choosing “no enzyme”, and then presses the “Submit a job” button. If one wishes to change the default mass error tolerance, it can be done under the “more parameter” toggle. One may also elect to include PTMs or deselect certain amino acids from consideration, those choices are available under the “Amino acids and PTMs” toggle. When using search methods that do not have a theoretical model for the score distribution or when the quality of the score model [11] is poor, one may wish to use a more conservative statistical significance assignment. In this case, a user may set 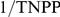 as the lower bound for the best 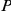-value for any given parent ion mass. This may help in preventing exaggerated/inappropriate statistical significance assignments.

### Generate score histogram: mode (ii)

**Figure 9:**
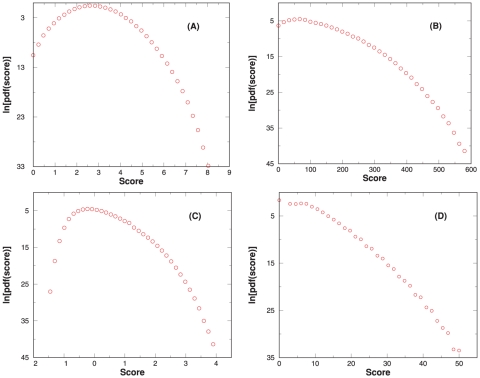
Example score PDF (normalized histogram) output by RAId_aPS. An MS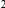 spectrum of parent ion mass 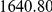 Da is queried with default parameters, and the resulting score PDF for RAId, K-score, XCorr, and Hyperscore are shown respectively in panels A, B, C, and D. The number of APP within 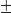 3Da of parent ion mass is about 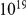.

Extraction of the statistical significance from a score distribution often requires a model, be it theoretically derived or empirically assumed, for the score distribution. One may test the robustness of a score model by examining how well the score model fits the database search score histograms. When using search methods that have a score model, one may first test how well the same score model applies when dealing with APP. If the score model loses stability, this may indicate that the score model is not robust in general. Given a query spectrum and a user-selected scoring function, RAId_aPS can be used to generate a score histogram of APP under the selected scoring scheme. Using an example spectrum, Figure 9 shows score histograms corresponding to the four scoring functions implemented in RAId_aPS.

### Reassign 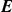-value : mode (iii)

Statistical significance inference from RAId_aPS only depends on the total number of qualified peptides inside the database searched but is not dependent on the peptide content inside the database. This is because RAId_aPS bases its statistics on the (weighted) score histogram obtained from scoring APP. As a consequence, without going through the database search again, RAId_aPS can be used to reassign statistical significance to a collection of candidate peptides. The candidate peptides may come from a flat list provided by the user, or they can also come from the output files of various search engines. RAId_aPS allows users to upload the output files from SEQUEST, X!Tandem, and Mascot for statistical significance reassignment.

**Figure 10:**
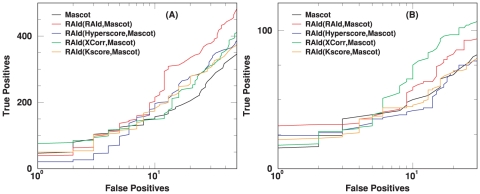
Example of reanalyzing output files from other search engine by combining with statistical significance assignment from RAId_aPS. In this example, we use the Mascot output files resulting from querying profile spectra (panel (A), the NHLBI data set) and centroid spectra (panel (B), A1–A4 of the ISB data set [28]) to the NCBI's nr database with proteins highly homologous to those that were present in the mixture removed. Since each data set is from a known mixture of proteins, it is possible to remove the proteins homologous to the true positives from the nr database. We then combine the calibrated 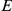-value [4] of Mascot with the 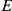-value obtained from RAId_aPS when either RAId score, Hyperscore, K-score or XCorr is used.

Although scoring functions similar to XCorr, K-score and Hyperscore have been implemented in RAId_aPS, other search engines' scoring functions might not be suitable for score histogram construction using dynamic programming. In this case, the user may wish to compare the statistical significance reported by a search engine with what is reported by RAId_aPS and even combine these reported significances. As an example of this usage and to test RAId_aPS's performance, we use as queries 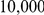 profile spectra (the NHLBI data set) as well as 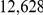 centroid spectra (A1–A4 of the ISB data set), each produced from a known mixture of target proteins. Using Mascot as the search engine, we searched in the NCBI's nr database with proteins highly homologous to the target proteins removed [11]. The output files were analyzed to produce ROC curves, the black solid curves in Figure 10. We then reanalyzed the candidate peptides' statistical significance by combining the statistical significance reported by Mascot with that reported by RAId_aPS using one additional scoring function. For both profile and centroid spectra, when combined with either the RAId score, K-score, or XCorr, one may obtain a retrieval performance that is comparable with or slightly better than that from Mascot alone (see Figure 10).

Since all the implemented scoring functions are accessible from RAId_aPS, one can score any new PTM peptide using any of the scoring functions available to RAId_aPS even when the original program does not yet include the PTMs of interest. This way, annotated PTM found by RAId_DbS [23] may be confirmed with other scoring functions in a natural manner and one may even combine the statistical significance as described below to increase the sensitivity in finding annotated PTMs and single amino acid polymorphisms (SAP).

## Discussion

In this section we will discuss another proposed use of the APP statistics in confidence assignment, remark on the effectiveness of combining search results using a different measure than ROC, propose avenues for improvement, and describe future directions.

When combined with database searches, the score histogram obtained by RAId_aPS also provides two useful quantities. First, it gives us the best peptide score 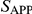 among APP. Although we did not pursue this way, it has been advocated that the difference between 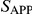 and the best database hit score per spectrum may serve as a statistical significance measure for the highest-scoring peptide hits found in the database [24]. Second, the score histogram provides us with 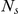, the (weighted) number of APP with score better than or equal to 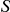. This number 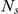 may also be used in conjunction with the (relative) difference between 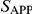 and the best database search score *per spectrum* while constructing statistical significance measures other than 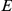-value.

A natural question to ask is: how much retrieval gain can one anticipate if one combines multiple scoring functions? Since FDR has been among the most popular metrics for assessing the performance, we briefly investigate this issue using FDR. Employing a frequently used procedure [31], we used the reverse *Homo sapiens* protein database as the decoy database to estimate the number of false positives and hence the FDR, by searching target database and decoy database separately for each query spectrum. All 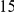 possible combinations of the four scoring functions available in RAId_aPS are tested using the data set PRIDE_Exp_mzData_Ac_8421.xml (containing 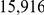 spectra), downloaded from the PRoteomics IDEntifications (PRIDE) database (http:www.ebi.ac.ukprideppp2_links.do). The results are summarized in Table 2 along with the average behavior associated with using one to four scoring functions. Since it is known that performance of a search engine may vary when the data to be analyzed changes [32], we like to focus more on the average behavior rather than individual performance of a scoring function or any specific combination of scoring functions. Based on the average retrieval result of Table 2, we first observe that on average there is an overall retrieval increase at 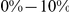 FDR rates when one combine two scoring functions versus using only one scoring function. We also note that there is an increase in retrieval performance at medium FDR rate when more scoring functions are combined. However, at very low FDR rates, it seems that combining more than two scoring functions stop helping the retrieval. Apparently, the performance boost does not continue indefinitely as more scoring functions are included. This is evidenced by an observable performance decline at low FDR rate when one combine all four scoring functions and compared to combine only three. The saturation of performance gain is reasonable if one takes into account the fact that most scoring functions seek similar evidences, the scope covered by combining more scoring functions can't keep increasing indefinitely.

**Table 2:** Example retrieval tests based on FDR.

Combination	FDR cutoff 0%	FDR cutoff 2.5%	FDR cutoff 5.0%	FDR cutoff 10%
R	377	822	856	948
K	83	709	790	977
H	568	775	849	908
X	467	821	885	996
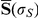	**373 (182)**	**781 (57)**	**845 (34)**	**957 (39)**
RK	485	956	1127	1654
RH	925	1143	1599	2375
RX	871	1024	1140	1574
KH	528	1019	1210	1679
KX	588	860	964	1146
HX	895	1064	1205	1532
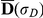	**715 (186)**	**1011 (87)**	**1207 (196)**	**1660 (365)**
RKH	485	849	2689	5328
RKX	474	792	1074	2425
RHX	725	867	1942	4795
KHX	443	658	910	1691
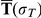	**531 (116)**	**791 (86)**	**1653 (716)**	**3559 (1537)**
RKHX ( 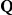 )	**332**	**662**	**1336**	**4148**

All 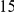 possible combinations of the four scoring functions available in RAId_aPS are shown along with the average behavior associated with using one to four scoring functions. The dataset PRIDE_Exp_mzData_Ac_8421.xml is used. The first column documents various combinations of scoring functions with the following abbreviations: R for RAId, K for K-score, H for hyperscore, and X for XCorr. The rest of the columns display the number of peptides identified at the false positive rate specified at the top of the column. The rows with bold characters indicate the average behavior of using a single (
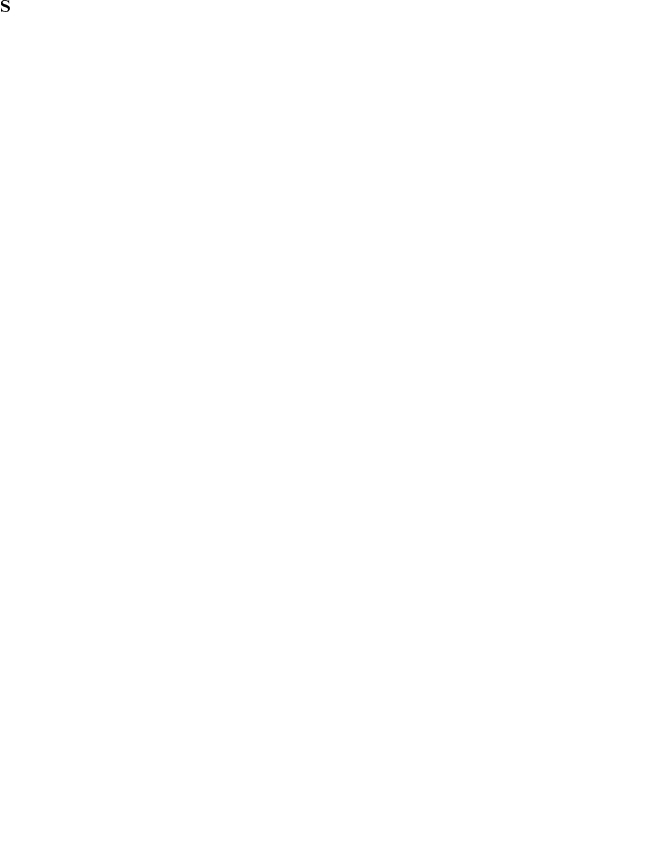
) scoring function, combining two (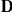) scoring functions, combining three (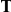) scoring functions, and combining four (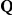) scoring functions. Within these rows, except the last one where only one combination possible, the standard deviation associated with each average is shown inside the parentheses to the right of the average.

By integrating existing annotated information into organismal databases, RAId_DbS is now able to incorporate during its data analysis annotated information such as SAP, PTM, and their disease associations if they exist [23]. This feature enables users to identify/include known polymorphisms/modifications in their searches without needing to blindly allow all possible SAPs and PTMs first and then post process to look up the literature/databases for explanations. Since all the implemented scoring functions of RAId_aPS are now within the same framework, we can let each plug-in scoring function incorporate in its scoring the new SAP/PTM peptides. This way, annotated SAP/PTM found by RAId_DbS may be confirmed by other implemented scoring approaches in a natural manner and one may even combine the statistical significances as described earlier to increase the sensitivity in finding annotated SAPs/PTMs.

In the near future, we also plan to include more scoring functions in RAId_aPS if their presence would enhance the retrieval performance without sacrifice statistical accuracy. For example, we will investigate the effect of a new scoring function, the compound Poisson. This is a natural way to incorporate intensity information into Poisson count statistics. The other scoring approach we will investigate is to deconvolute the peptide length information. The reason to consider this alternative arises from the observation that many scoring functions introduce different heuristics to correct for the scores associated with candidate peptides of different lengths. The purpose of these peptide length correction factors is to balance the fact that longer peptides are likely to find more evidence peaks and thus the collected evidence scores may require some length correction in order to make the comparison among peptides of various lengths impartial. If we group peptides of the same lengths and obtain statistical significance separately for peptide candidates of each length, we no longer need to introduce any length correction factor. This approach is not feasible for regular database searches since the sample size of peptides of a fixed length may be too small. For our APP scheme, however, we always have a large number of peptides participating in our score histogram even if the peptide length is fixed. Therefore, the idea of deconvoluting the peptide lengths becomes feasible for RAId_aPS.

## Supporting Information

Text S1 **Spectral Filtering and Scoring Functions.** The main objective of this supplementary text is to document what we found from the source codes of various search methods about their spectral filtering strategies. Although effort is invested to faithfully reproduce these filtering strategies, we do no intend to provide a logical explanation of these filtering methods. Readers interested in obtaining logical explanations of these strategies should contact the original code authors. There also exist other heuristics in various scoring functions that we chose to ignore. As shown in Figure 6 and Figure S6, and in dashed curves of Figures S8 and S9, the performance of these scoring functions without heuristics do not suffer from poorer retrieval compared to their original implementations with heuristics included. (PDF)

Figure S1 **Filtering accuracy assessment.** For every raw spectrum, one generates six filtered spectra:three associated with Hyperscore/XCorr/K-score implemented in RAId aPS and the other three respectively produced by X!Tandem/Crux/X!Tandem(with K-score plug-in). The mass fragments of every filtered spectrum are then read to a mass grid. The spectrum is then viewed as a vector with non-vanishing components only at the component/mass indices populated. One then normalizes each *filtered* spectrum vector into unit length. An inner product of any two filtered spectral vectors represents the correlation between them. When the spectral quality does not pass a method-dependent threshold, the corresponding filtering protocol may turn the raw spectrum into a null spectrum without further searching the database. Therefore the total number of spectra passing through the filtering stage might be smaller than the total number of raw spectra, which is also reected in the histograms. Two sets of data are used for this evaluation. The centroid data, consisting of 38; 424 spectra, are from the ISB data set [1]. The pro_le data, consisting of 10; 000 spectra, are from the NHLBI data set [2]. Panel A(D) shows the histogram of correlation between the RAId aPS K-score and the X!Tandem K-score plug-in using centroid(profile) data. Panel B(E) shows the histogram of correlation between the RAId aPS XCorr and the Crux XCorr using centroid(profile) data. Panel C(F) shows the histogram of correlation between the RAId aPS Hyperscore and the X!Tandem Hyperscore using centroid(profile) data. The correlation strength being always one means that RAId aPS is able to faithfully reproduce the filtering strategies originally designed for Hyperscore, XCorr, and K-score. (PDF)

Figure S2 **Histograms of correlations between filtering strategies.** This Figure is the same as Figure 3 except that the 10, 000 raw spectra used are profile data from the NHLBI data set [1]. (PDF)

Figure S3 **Score correlations.** A subset of the NHLBI profile data set [1] was used to perform this evaluation. For each scoring function, when the best hit per spectrum (analyzed using the analysis program that the scoring function was originally used for) is a true positive, that candidate peptide is scored again using the corresponding scoring function implemented in RAId aPS. Each true positive best hit thus gives rise to two scores and plotted using the following rule: the first score is used as the ordinate while the second score (from RAId aPS) is used as the abscissa. Including 500 spectra, panel A is for the RAId score. Panel B is for Hyperscore and contains 495 spectra. The result of K-score is shown in panel C with 310 spectra. Shown with 500 spectra, panel D documents the results for XCorr. (PDF)

Figure S4 **E-value accuracy assessment.** The agreement between the reported E-value and the textbook definition is examined using profile data (panel (A–B), 10, 000 spectra of the NHLBI data set) as well as centroid data (panel (C–D), A1–A4 subsets of ISB data set). The NCBI's nr (of size 500 MB) database with true positives removed is used for this assessment. The molecular weight range considered while searching the database is [MW − ,MW + ]. In each panel, the dashed lines, corresponding to x  =  5y and x  =  y/5, are used to provide a visual guide regarding how close/off the experimental curves are from the theoretical curve. (PDF)

Figure S5 **E-value accuracy assessment.** The agreement between the reported E-value and the textbook definition is examined using profile data (the NHLBI data set: 10, 000 spectra). The random database size used is 500 MB. The molecular weight range considered while searching the database is [MW − , MW + ]. In each panel, the dashed lines, corresponding to x  =  5y and x  =  y/5, are used to provide a visual guide regarding how close/off the experimental curves are from the theoretical curve. (PDF)

Figure S6 **ROC curves for the profile data (NHLBI data set** [**1**] **).** For each of the four scoring functions considered, a set of ROC curves is shown. These ROC curves include the results from running the designated program associated with that scoring function, the results from running RAId aPS in the database search mode, and the results from combining with each one of the three other scoring functions. Panel (A) shows the results from RAId score, whose designated program is RAId DbS. Panel (B) displays the results from K-score, whose designated program is X!Tandem. Panel (C) exhibits the results from XCorr, which is mostly employed by SEQUEST. Panel (D) presents the results from Hyperscore, whose designated program is also X!Tandem. (PDF)

Figure S7 **ROC curves for the profile data (NHLBI data set** [**1**] **) when considering only the best hit per spectrum.** For each of the four scoring functions considered, a set of ROC curves is shown. These ROC curves include in the consideration only the best hit per spectrum from running the designated program associated with that scoring function, the best hit per spectrum from running RAId aPS in the database search mode, and the best hit per spectrum from combining with each of the three other scoring functions. Panel (A) shows the results from RAId score, whose designated program is RAId DbS. Panel (B) displays the results from K-score, whose designated program is X!Tandem. Panel (C) exhibits the results from XCorr, which is mostly employed by SEQUEST. Panel (D) presents the results from Hyperscore, whose designated program is also X!Tandem. (PDF)

Figure S8 **ROC curves when highly homologous proteins** [**1**] **are also counted as true positive proteins.** Plots done this way are analogous to the ROC plots obtained using a decoy database to estimate the number of false positives. Each panel displays the results of a scoring function. The resulting ROC curves from using RAId aPS implementation and the implementation in the original search program are both shown. The results from profile data (NHLBI data set [2]) are shown in solid curves, while the results from centroid data (A1–A4 of ISB data set [3]) are shown in long-dash curves. Panels (A,B,C,D) respectively display the results from using RAId score, K-score, XCorr, and Hyperscore. Except for RAId score, the RAId aPS implemented scoring functions performs comparably to the original implementation in other search methods. (PDF)

Figure S9 **ROC curves when highly homologous proteins** [**1**] **are removed from the nr database and thus are not counted towards true positives or false positives.** Each panel displays the results of a scoring function. The resulting ROC curves from using RAId aPS implementation and the original implementation in other search program are both shown. The results from profile data (NHLBI dataset [2]) are shown in solid curves, while the results from centroid data (A1–A4 of ISB data set [3]) are shown in long-dash curves. Panels (A,B,C,D) respectively display the results from using RAId score, K-score, XCorr, and Hyperscore. The RAId aPS implemented scoring functions performs comparably to the original implementation in other search methods. (PDF)
