# Supplementary material for: RAId_aPS: MS/MS Analysis with Multiple Scoring Functions and Spectrum-Specific Statistics
Source: PLoS One. 2010 Nov 16;5(11):e15438. doi: 10.1371/journal.pone.0015438 (PMC2982831; doi:10.1371/journal.pone.0015438)
Supplement: Text S1 — Spectral Filtering and Scoring Functions. The main objective of this supplementary text is to document what we found from the source codes of various search methods about their spectral filtering strategies. Although effort is invested to faithfully reproduce these filtering strategies, we do no intend to provide a logical explanation of these filtering methods. Readers interested in obtaining logical explanations of these strategies should contact the original code authors. There also exist other heuristics in various scoring functions that we chose to ignore. As shown in Figure 6 and Figure S6, and in dashed curves of Figures S8 and S9, the performance of these scoring functions without heuristics do not suffer from poorer retrieval compared to their original implementations with heuristics included. (PDF) [file pone.0015438.s001.pdf]

Text S1: Spectral Filtering and Scoring Functions

The main objective of this supplementary text is to document what we found from the source codes of various search methods about their spectral filtering strategies. Although effort is invested to faithfully reproduce these filtering strategies, we do no intend to provide a logical explanation of these filtering methods. Readers interested in obtaining logical explanations of these strategies should contact the original code authors. There also exist other heuristics in various scoring functions that we chose to ignore. As shown in Figure 6 and Figure S6, and in dashed curves of Figures S8 and S9, the performance of these scoring functions without heuristics do not suffer from poorer retrieval compared to their original implementations with heuristics included.

Notation

Before we begin documentation of the filtering strategies associated with different scoring functions as well as our implementation of these scoring functions, we define a set of notations.

`mw` precursor ion molecular weight at charge +1 state
z peptide/fragment charge state
m/z mass over charge
m_i molecular mass of fragment i in the MS/MS spectrum
Δm_i mass disagreement between the theoretical mass of fragment i and m_i.
I_i peak intensity of an observed m_i
`hw` highest observed molecular weight
`lw` lowest observed molecular weight
π a peptide sequence
δm MS/MS spectrum m/z accuracy
Da Dalton
$T(\pi)$ represents the set of theoretical peaks used for scoring or the total number of items in that set.
$l(\pi)$ length of peptide π, the total number of amino acids in peptide π.
H molecular weight of a hydrogen atom

RAId Score Filtering and Scoring Function

1) The details of RAId score filtering are explained in RAId_DbS original publication [1].

2) The RAId scoring function (also used in RAId_DbS) is define as

$$\text{RAId } S(\pi) = \frac{1}{T(\pi)} \sum_{i=1}^{T(\pi)} \ln(I_i)\, e^{-\Delta m_i} \theta(1 - \Delta m_i),$$

with the default theoretical peaks for scoring $T(\pi) = \{b_n, y_n\}_{n=1}^{l(\pi)-1}$ and with $l(\pi)$ representing the number of amino acids in peptide π.

X!Tandem Filtering and Hyperscore

1) m/z fragments that are within ± 0.95 Da of each other are removed from the MS/MS spectrum. When two fragments are within ± 0.95 Da of each other the fragment with the highest intensity is kept.

```
for(i = lw; i < hw; i = i+1)
   for(j = lw+1; j ≤ hw; j = j+1)
     do
     if ((m_i - m_j) < 0.95   &&   I_i < I_j )  m_i = 0 ;   I_i = 0 ;
```

2) m/z fragments that are in the mass range $(x - 5/z, x + 5/z)$ are removed from the MS/MS spectrum, where $x = 1.00727 + (\texttt{mw} - 1.00727)/z$.

3) m/z fragments that are lighter than 150 Da are removed from the MS/MS spectrum.

4) The filtered spectrum is normalized to have maximum intensity 100 and fragments with normalized intensity less than 1 are removed from the spectrum.

5) Try to determine if the spectrum is purely noise.

```
if(z=1 || z=2)  x = mw - 600
else   x = mw/z
if( Heaviest Filtered Fragment  < x)
   exit  (spectrum is noisy)
else if( Total Number of Filtered Fragments < 5)
   exit  (spectrum is noisy)
```

6) m/z fragments that are within ± 1.5 Da are removed from the spectrum. When two fragments are within ± 1.5 Da of each other the fragment with the highest intensity is kept, as shown in step 1).

7) The final filtered spectrum consists at most 50 fragments having the highest intensities.

8) The molecular weights of the fragments in the filtered spectrum are transformed to integer values using the MS/MS spectrum mass accuracy (δm).

$m_i = \texttt{int}[\frac{m_i}{\delta m} + 0.5]$

9) After the transformation above, the mass indices to either sides of m_i are initialized as follows:

```
for(i=1; i < int [mw /δm]; i = i+1)
   do
   if( I_{i-1} < I_i) I_{i-1} = I_i
   if( I_{i+1} < I_i) I_{i+i} = I_i
```

*Note: To speed up the code in RAId_aPS implementation the intensity is further scale by multiplying it by a factor of 0.1: $I_i = 0.1 \times I_i$.

10) Theoretical fragments chosen for scoring $T(\pi) = \{b_n, y_n\}_{n=1}^{l(\pi)-1}$. For a precursor ion with

charge $z = 2$, the score is give by:

$$\text{Hyperscore } S(\pi) = 4\log_{10}\left[10\left(\sum_{i=1}^{T(\pi)} I_i\right) b\,!\; y!\right]$$

The multiplication factor of "10" in the above scoring function is introduced because RAId_aPS scaled the intensity by a factor of 0.1 as mentioned above. To keep RAId_aPS's run time reasonable, for parent ion in higher charge state, RAId_aPS scoring deviates from the X!Tandem Hyperscore. Basically, counter $b(y)$ totals the number of b-(y-)type of evidence peaks without separating them further into different charge states.

Crux Filtering and XCorr

1) The intensities present in the MS/MS spectrum are transformed by taking the square root of the original intensities.

$$I_i = \sqrt{I_i}$$

2) m/z fragments that are in the mass range $(x - 15, x + 15)$ are removed from the MS/MS spectrum, where $x = (\texttt{mw} + z - 1.0)/z$.

3) m/z fragments that are greater than x are removed from the MS/MS spectrum, where $x = (\texttt{mw} + z - 1 + 50)$.

4) Observed m/z fragments in the MS/MS spectrum are transformed to integer values using a mass grid where neighboring points are spaced by 1.0005079 Da.

```
x = (mw + z - 1 + 50)
for(i = 0; i ≤ x; i = i+1) s[i]=0
for(i = 0; i ≤ x; i = i+1)
   do
   m_i = int[m_i/1.0005079 + 0.5]
   if(s[m_i]< I_i) s[m_i] = I_i
```

5) The MS/MS spectrum's m/z range is divided into 10 mass regions. The width of each region is equal to the heaviest observed fragment molecular weight hw divided by 10.
$mr = \texttt{int}[\texttt{hw}/10]$
The 10 regions are: $[0, mr), [mr, 2mr), [2mr, 3mr), \ldots,$ and $[9mr, 10mr)$

6) The peak intensities within each region, if m/z fragments exist, are normalized to have maximum intensity 50. If no mass fragment is present in a given region, the maximum region intensity is set to 0.

7) The final filtered spectrum is obtained by applying the following operation to the spectrum intensities

```
x = (mw + z - 1 + 50)
for( i = 0; i < x; i = i+1
   do  for(v = 0, j = i-75; j ≤ i+75; j++)
          do  if(j ≥0 &&  j < x)  v = v + s[j]
   I_i = s[i] - v/150
```

8) The XCorr score is computed by taking the dot product between the theoretical fragments $T(\pi)$ and the filtered spectrum I_i. The default series used for scoring is $T(\pi) = \{b_n, y_n, b_n - H, b_n + H, y_n - H, y_n + H, b_n - H_2O, b_n - NH_3, y_n - NH_3, a_n\}_{n=1}^{l(\pi)-1}$, with each series contributing to the score respectively weighted by $w_i = \{50, 50, 25, 25, 25, 25, 10, 10, 10, 10\}$.

$$\text{XCorr } S(\pi) = \frac{1}{10000} \sum_{i=1}^{T(\pi)} w_i I_i \ .$$

Note: To speed up the code, RAId_aPS implements XCorr with different scales for intensity and weight factors. First, the peak intensity is scale down by a factor of ten: $I_i = 0.1 \times I_i$. Second, the weight factors are scaled down by a factor of 50, thus $w_i = \{1, 1, 0.5, 0.5, 0.5, 0.5, 0.2, 0.2, 0.2, 0.2\}$. Since we are absorbing a factor 1/50 into the weights and another factor 1/10 into the peak intensities, the Xcorr score in RAId_aPS reads

$$\text{XCorr } S(\pi) = \frac{1}{20} \sum_{i=1}^{T(\pi)} w_i I_i \ ,$$

because the factor 1/10000 can be written as

$$\frac{1}{10000} = \frac{1}{50} \times \frac{1}{10} \times \frac{1}{20} \ .$$

K-score Filtering and Scoring Function

1) The intensities present in the MS/MS spectrum are transformed by taking the square root of the original intensities.

$$I_i = \sqrt{I_i}$$

2) m/z fragments less than x are removed from the MS/MS spectrum, where $x = (\texttt{mw} + (z-1) * 1.00075) \times 2/z + 10.5$.

3) The observed m/z fragments in the MS/MS spectrum are transformed to integer values using a mass grid where neighboring points are spaced by 1.0005 Da.

```
for(i = 0; i ≤ (mw+128); i = i+1) s[i]=0
for(i = 0; i ≤ hw; i = i+1)
    do
    m_i = int [m_i/1.0005 + 0.5]
    if (s[m_i]< I_i)  s[m_i] = I_i
```

4) The spectrum's m/z range is partitioned into intervals with the number of intervals depending on the value of R, see below.

```
x = (mw + (z-1)*1.00075)× 2/z + 10.5
R = min(x,hw+10)-lw
```

The number of partitions N(R) is determined by the condition below

$$\text{N}(R) = \begin{cases} 10 & : \ R > 3000 \\ 9 & : \ R > 2500 \\ 8 & : \ R > 2000 \\ 7 & : \ R > 1500 \\ 6 & : \ R > 1000 \\ 5 & : \ R > 0 \end{cases}$$

5) Within each partition the spectrum is scaled such that the maximum peak intensity in each interval equals to the maximum intensity in the MS/MS spectrum right after step 1). Peaks with intensities that are less than 5 percent of the maximum spectrum intensity are removed.

6) The spectrum is normalized to a unit vector.

```
for(x = 0, i = 0; i ≤ hw; i = i+1) x = x+s[i]× s[i]
for(i = 0; i ≤ hw; i = i+1) s[i] = s[i]/√x
```

7) The final filtered spectrum is obtained by applying the following transformation to the peak intensities

```
for(i = 0; i ≤ hw; i = i+1)
    do  for(v = 0, j = i-50; j ≤ i+50; j++)
            do  if(j ≥ 0  &&  j ≤ hw)  v = v+s[j]
    if (s[i]- v/101 > 0 )
            I_i = s[i] - v/101
```

8) The K-score is computed by taking the dot product between the theoretical fragments $T(\pi)$ and the filtered spectrum I_i. The default fragmentation series used for scoring are $T(\pi) = \{b_n, y_n, b_n - H, b_n + H, y_n - H, y_n + H\}_{n=1}^{l(\pi)-1}$, with each series contributing to the score respectively weighted by $w_i = \{1, 1, 0.5, 0.5, 0.5, 0.5\}$.

$$\text{K–Score} \quad S(\pi) = \frac{1000 \ln(l)}{3\sqrt{l}} \sum_{i=1}^{T(\pi)} w_i I_i$$

References

1. Alves G, Ogurtsov AY, Yu YK (2007) RAId_DbS: Peptide identification using database searches with realistic statistics. Biology Direct 2: 25.
