# Supplementary material for: RAId_aPS: MS/MS Analysis with Multiple Scoring Functions and Spectrum-Specific Statistics
Source: PLoS One. 2010 Nov 16;5(11):e15438. doi: 10.1371/journal.pone.0015438 (PMC2982831; doi:10.1371/journal.pone.0015438)
Supplement: Figure S1 — Filtering accuracy assessment. For every raw spectrum, one generates six filtered spectra:three associated with Hyperscore/XCorr/K-score implemented in RAId aPS and the other three respectively produced by X!Tandem/Crux/X!Tandem(with K-score plug-in). The mass fragments of every filtered spectrum are then read to a mass grid. The spectrum is then viewed as a vector with non-vanishing components only at the component/mass indices populated. One then normalizes each filtered spectrum vector into unit length. An inner product of any two filtered spectral vectors represents the correlation between them. When the spectral quality does not pass a method-dependent threshold, the corresponding filtering protocol may turn the raw spectrum into a null spectrum without further searching the database. Therefore the total number of spectra passing through the filtering stage might be smaller than the total number of raw spectra, which is also reected in the histograms. Two sets of data are used for this evaluation. The centroid data, consisting of 38; 424 spectra, are from the ISB data set [1]. The pro_le data, consisting of 10; 000 spectra, are from the NHLBI data set [2]. Panel A(D) shows the histogram of correlation between the RAId aPS K-score and the X!Tandem K-score plug-in using centroid(profile) data. Panel B(E) shows the histogram of correlation between the RAId aPS XCorr and the Crux XCorr using centroid(profile) data. Panel C(F) shows the histogram of correlation between the RAId aPS Hyperscore and the X!Tandem Hyperscore using centroid(profile) data. The correlation strength being always one means that RAId aPS is able to faithfully reproduce the filtering strategies originally designed for Hyperscore, XCorr, and K-score. (PDF) [file pone.0015438.s002.pdf]

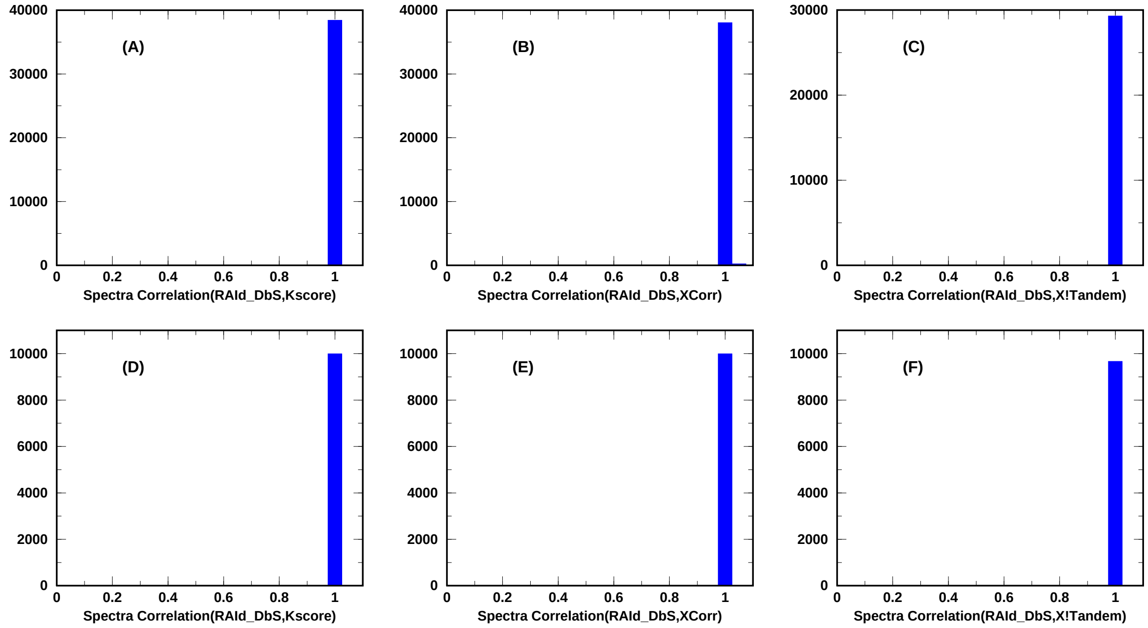

Figure S1. Filtering accuracy assessment. For every raw spectrum, one generates six filtered spectra: three associated with Hyperscore/XCorr/K-score implemented in RAId_aPS and the other three respectively produced by X!Tandem/Crux/X!Tandem(with K-score plug-in). The mass fragments of every filtered spectrum are then read to a mass grid. The spectrum is then viewed as a vector with non-vanishing components only at the component/mass indices populated. One then normalizes each *filtered* spectrum vector into unit length. An inner product of any two filtered spectral vectors represents the correlation between them. When the spectral quality does not pass a method-dependent threshold, the corresponding filtering protocol may turn the raw spectrum into a null spectrum without further searching the database. Therefore the total number of spectra passing through the filtering stage might be smaller than the total number of raw spectra, which is also reflected in the histograms. Two sets of data are used for this evaluation. The centroid data, consisting of $38,424$ spectra, are from the ISB data set [1]. The profile data, consisting of $10,000$ spectra, are from the NHLBI data set [2]. Panel A(D) shows the histogram of correlation between the RAId_aPS K-score and the X!Tandem K-score plug-in using centroid(profile) data. Panel B(E) shows the histogram of correlation between the RAId_aPS XCorr and the Crux XCorr using centroid(profile) data. Panel C(F) shows the histogram of correlation between the RAId_aPS Hyperscore and the X!Tandem Hyperscore using centroid(profile) data. The correlation strength being always one means that RAId_aPS is able to faithfully reproduce the filtering strategies originally designed for Hyperscore, XCorr, and K-score.

References

1. Keller A, Samuel P, Nesvizhskii AI, Stolyar S, Goodlett DR, et al. (2002) Experimental protein mixture for validating tandem mass spectral analysis. OMICS 6: 207-212.

2. Alves G, Ogurtsov AY, Wu WW, Wang G, Shen RF, et al. (2007) Calibrating E-values for MS^2 library search methods. Biology Direct 2: 26.
