# Supplementary material for: RAId_aPS: MS/MS Analysis with Multiple Scoring Functions and Spectrum-Specific Statistics
Source: PLoS One. 2010 Nov 16;5(11):e15438. doi: 10.1371/journal.pone.0015438 (PMC2982831; doi:10.1371/journal.pone.0015438)
Supplement: Figure S2 — Histograms of correlations between filtering strategies. This Figure is the same as Figure 3 except that the 10, 000 raw spectra used are profile data from the NHLBI data set [1]. (PDF) [file pone.0015438.s003.pdf]

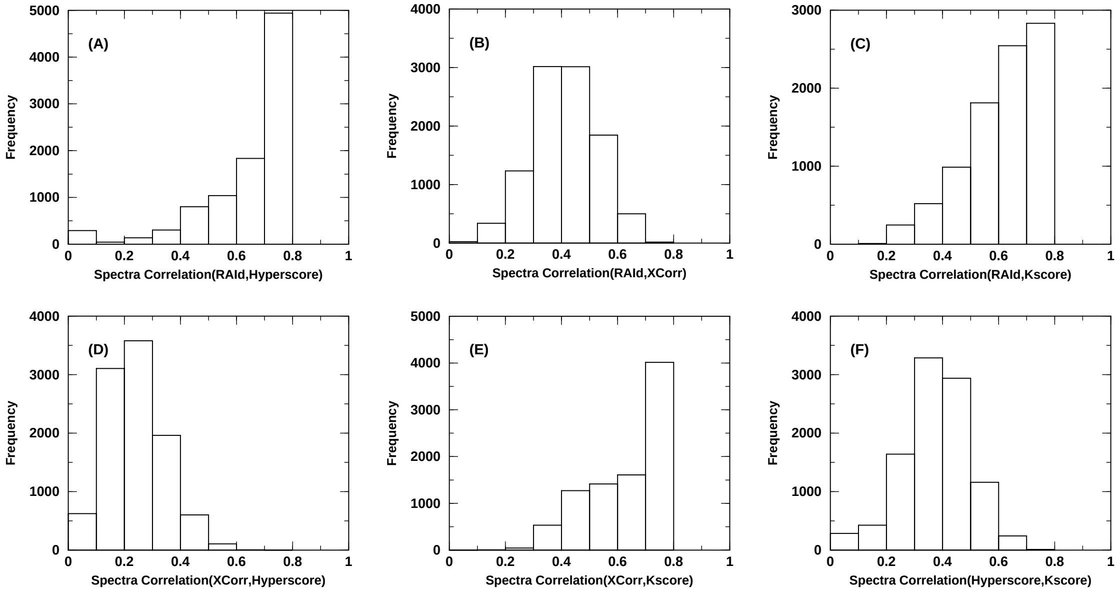

Figure S2. Histograms of correlations between filtering strategies. This Figure is the same as Figure 3 except that the $10,000$ raw spectra used are profile data from the NHLBI data set. [1]

References

1. Alves G, Ogurtsov AY, Wu WW, Wang G, Shen RF, et al. (2007) Calibrating E-values for MS^2 library search methods. Biology Direct 2: 26.
