# Supplementary material for: RAId_aPS: MS/MS Analysis with Multiple Scoring Functions and Spectrum-Specific Statistics
Source: PLoS One. 2010 Nov 16;5(11):e15438. doi: 10.1371/journal.pone.0015438 (PMC2982831; doi:10.1371/journal.pone.0015438)
Supplement: Figure S3 — Score correlations. A subset of the NHLBI profile data set [1] was used to perform this evaluation. For each scoring function, when the best hit per spectrum (analyzed using the analysis program that the scoring function was originally used for) is a true positive, that candidate peptide is scored again using the corresponding scoring function implemented in RAId aPS. Each true positive best hit thus gives rise to two scores and plotted using the following rule: the first score is used as the ordinate while the second score (from RAId aPS) is used as the abscissa. Including 500 spectra, panel A is for the RAId score. Panel B is for Hyperscore and contains 495 spectra. The result of K-score is shown in panel C with 310 spectra. Shown with 500 spectra, panel D documents the results for XCorr. (PDF) [file pone.0015438.s004.pdf]

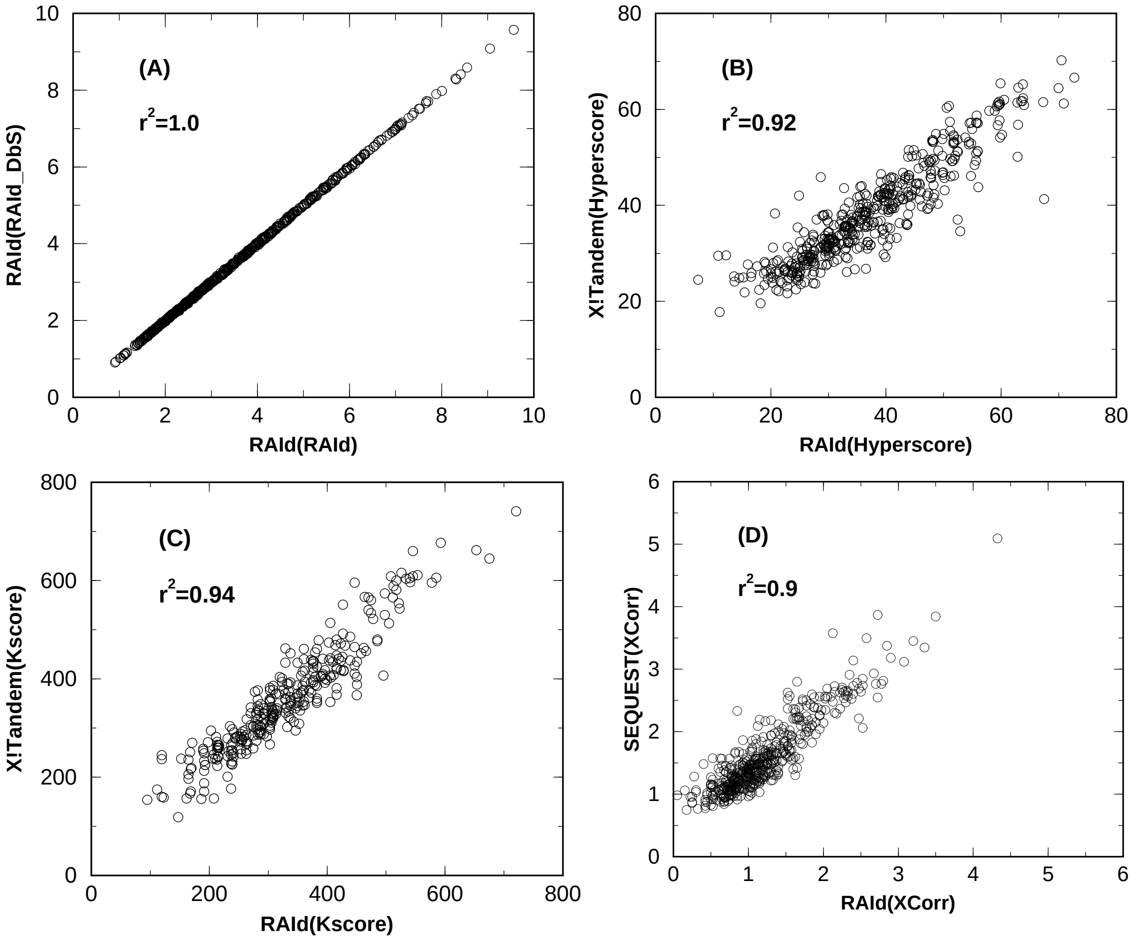

Figure S3. Score correlations. A subset of the NHLBI profile data set [1] was used to perform this evaluation. For each scoring function, when the best hit per spectrum (analyzed using the analysis program that the scoring function was originally used for) is a true positive, that candidate peptide is scored again using the corresponding scoring function implemented in RAId_aPS. Each true positive best hit thus gives rise to two scores and plotted using the following rule: the first score is used as the ordinate while the second score (from RAId_aPS) is used as the abscissa. Including 500 spectra, panel A is for the RAId score. Panel B is for Hyperscore and contains 495 spectra. The result of K-score is shown in panel C with 310 spectra. Shown with 500 spectra, panel D documents the results for XCorr.

References

1. Alves G, Ogurtsov AY, Wu WW, Wang G, Shen RF, et al. (2007) Calibrating E-values for MS^2 library search methods. Biology Direct 2: 26.
