# Supplementary material for: RAId_aPS: MS/MS Analysis with Multiple Scoring Functions and Spectrum-Specific Statistics
Source: PLoS One. 2010 Nov 16;5(11):e15438. doi: 10.1371/journal.pone.0015438 (PMC2982831; doi:10.1371/journal.pone.0015438)
Supplement: Figure S4 — E-value accuracy assessment. The agreement between the reported E-value and the textbook definition is examined using profile data (panel (A–B), 10, 000 spectra of the NHLBI data set) as well as centroid data (panel (C–D), A1–A4 subsets of ISB data set). The NCBI's nr (of size 500 MB) database with true positives removed is used for this assessment. The molecular weight range considered while searching the database is [MW − ,MW + ]. In each panel, the dashed lines, corresponding to x = 5y and x = y/5, are used to provide a visual guide regarding how close/off the experimental curves are from the theoretical curve. (PDF) [file pone.0015438.s005.pdf]

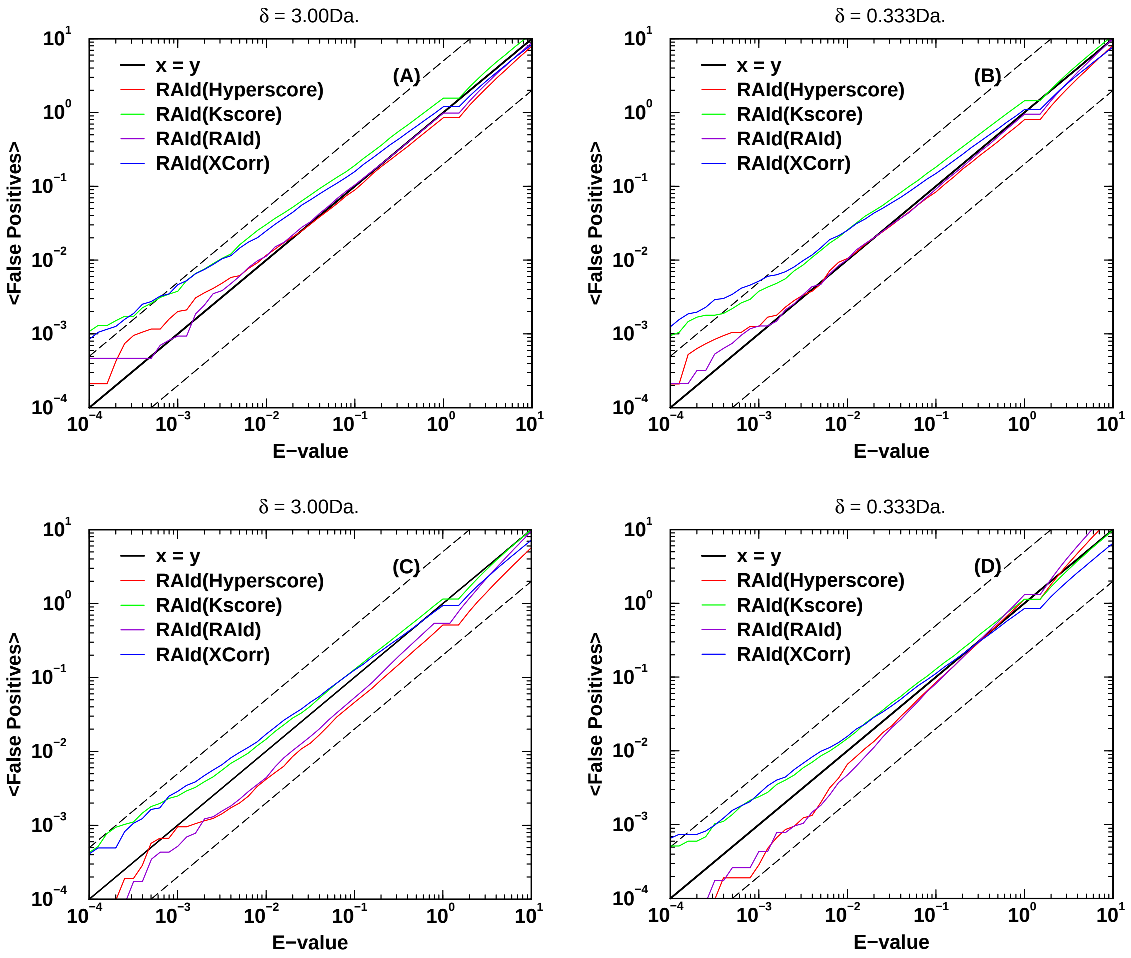

Figure S4. E-value accuracy assessment. The agreement between the reported E-value and the textbook definition is examined using profile data (panel (A-B), $10,000$ spectra of the NHLBI data set) as well as centroid data (panel (C-D), A1-A4 subsets of ISB data set). The NCBI's nr (of size 500 MB) database with true positives removed is used for this assessment. The molecular weight range considered while searching the database is $[MW - \delta, MW + \delta]$. In each panel, the dashed lines, corresponding to $x = 5y$ and $x = y/5$, are used to provide a visual guide regarding how close/off the experimental curves are from the theoretical curve.
