# Supplementary material for: RAId_aPS: MS/MS Analysis with Multiple Scoring Functions and Spectrum-Specific Statistics
Source: PLoS One. 2010 Nov 16;5(11):e15438. doi: 10.1371/journal.pone.0015438 (PMC2982831; doi:10.1371/journal.pone.0015438)
Supplement: Figure S5 — E-value accuracy assessment. The agreement between the reported E-value and the textbook definition is examined using profile data (the NHLBI data set: 10, 000 spectra). The random database size used is 500 MB. The molecular weight range considered while searching the database is [MW − , MW + ]. In each panel, the dashed lines, corresponding to x = 5y and x = y/5, are used to provide a visual guide regarding how close/off the experimental curves are from the theoretical curve. (PDF) [file pone.0015438.s006.pdf]

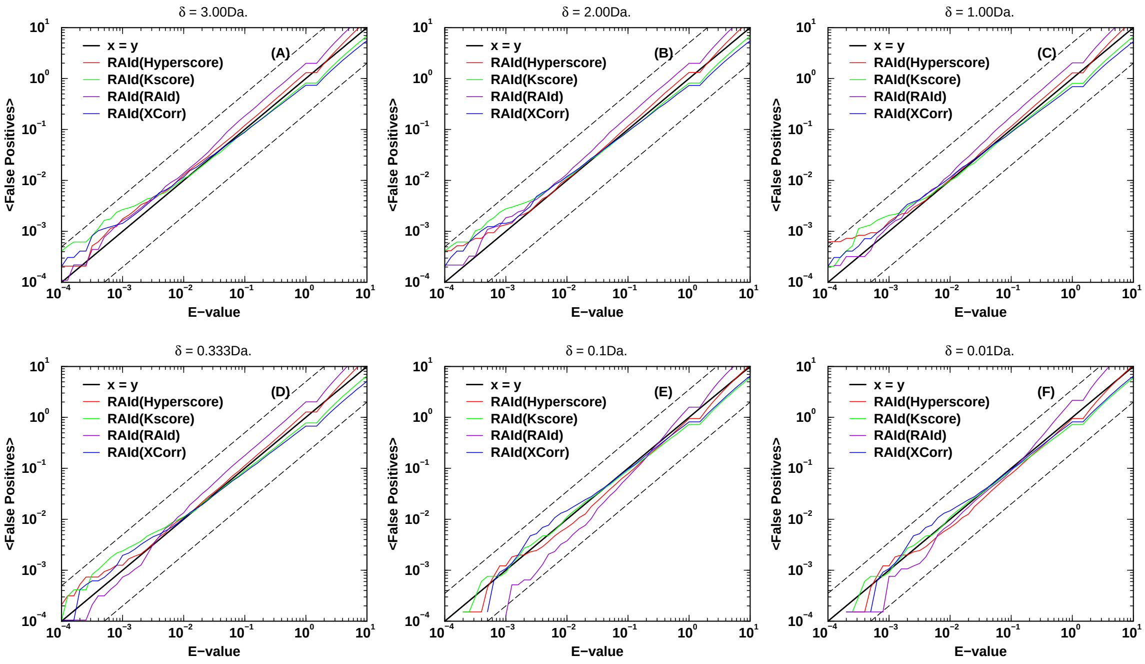

Figure S5. E-value accuracy assessment. The agreement between the reported E-value and the textbook definition is examined using profile data (the NHLBI data set: $10,000$ spectra). The random database size used is 500 MB. The molecular weight range considered while searching the database is $[MW - \delta, MW + \delta]$. In each panel, the dashed lines, corresponding to $x = 5y$ and $x = y/5$, are used to provide a visual guide regarding how close/off the experimental curves are from the theoretical curve.
