# Supplementary material for: RAId_aPS: MS/MS Analysis with Multiple Scoring Functions and Spectrum-Specific Statistics
Source: PLoS One. 2010 Nov 16;5(11):e15438. doi: 10.1371/journal.pone.0015438 (PMC2982831; doi:10.1371/journal.pone.0015438)
Supplement: Figure S7 — ROC curves for the profile data (NHLBI data set [1] ) when considering only the best hit per spectrum. For each of the four scoring functions considered, a set of ROC curves is shown. These ROC curves include in the consideration only the best hit per spectrum from running the designated program associated with that scoring function, the best hit per spectrum from running RAId aPS in the database search mode, and the best hit per spectrum from combining with each of the three other scoring functions. Panel (A) shows the results from RAId score, whose designated program is RAId DbS. Panel (B) displays the results from K-score, whose designated program is X!Tandem. Panel (C) exhibits the results from XCorr, which is mostly employed by SEQUEST. Panel (D) presents the results from Hyperscore, whose designated program is also X!Tandem. (PDF) [file pone.0015438.s008.pdf]

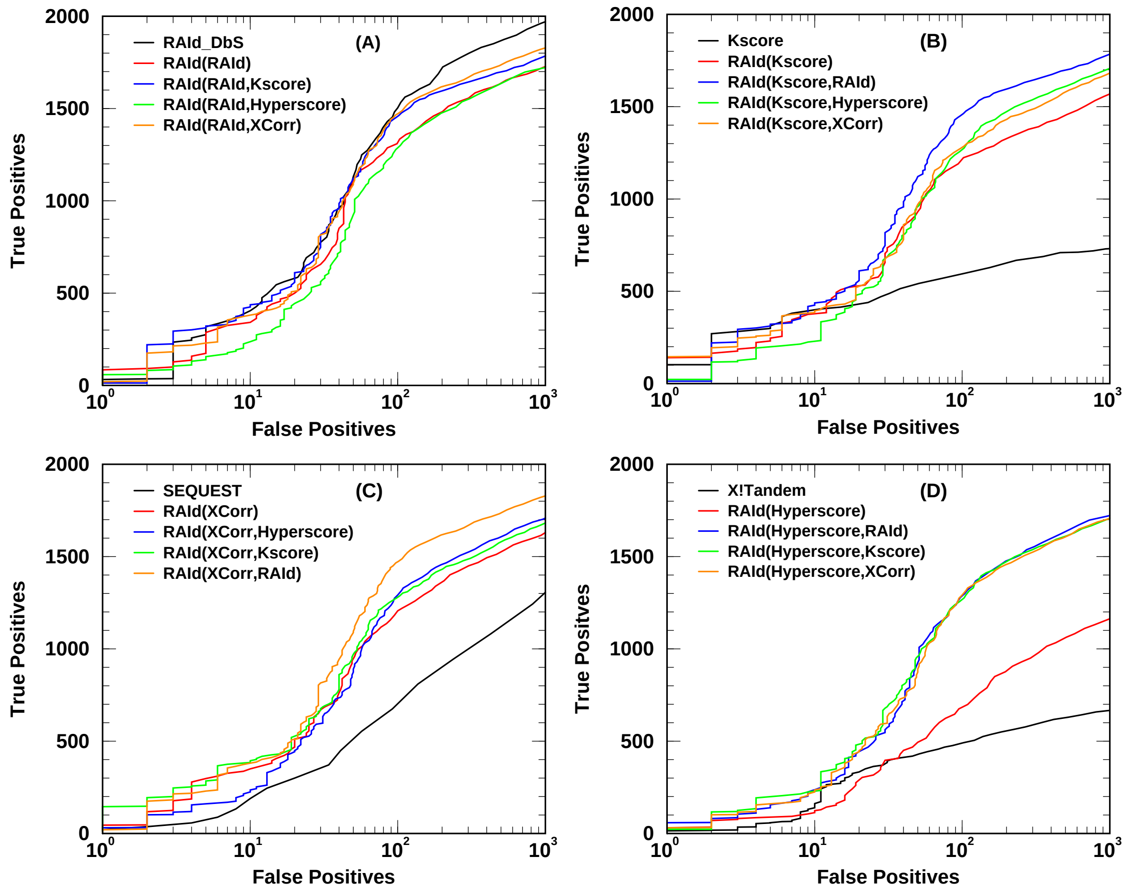

Figure S7. ROC curves for the profile data (NHLBI data set [1]) when considering only the best hit per spectrum. For each of the four scoring functions considered, a set of ROC curves is shown. These ROC curves include in the consideration only the best hit per spectrum from running the designated program associated with that scoring function, the best hit per spectrum from running RAId_aPS in the database search mode, and the best hit per spectrum from combining with each of the three other scoring functions. Panel (A) shows the results from RAId score, whose designated program is RAId_DbS. Panel (B) displays the results from K-score, whose designated program is X!Tandem. Panel (C) exhibits the results from XCorr, which is mostly employed by SEQUEST. Panel (D) presents the results from Hyperscore, whose designated program is also X!Tandem.

References

1. Alves G, Ogurtsov AY, Wu WW, Wang G, Shen RF, et al. (2007) Calibrating E-values for MS^2 library search methods. Biology Direct 2: 26.
