# Supplementary material for: RAId_aPS: MS/MS Analysis with Multiple Scoring Functions and Spectrum-Specific Statistics
Source: PLoS One. 2010 Nov 16;5(11):e15438. doi: 10.1371/journal.pone.0015438 (PMC2982831; doi:10.1371/journal.pone.0015438)
Supplement: Figure S9 — ROC curves when highly homologous proteins [1] are removed from the nr database and thus are not counted towards true positives or false positives. Each panel displays the results of a scoring function. The resulting ROC curves from using RAId aPS implementation and the original implementation in other search program are both shown. The results from profile data (NHLBI dataset [2]) are shown in solid curves, while the results from centroid data (A1–A4 of ISB data set [3]) are shown in long-dash curves. Panels (A,B,C,D) respectively display the results from using RAId score, K-score, XCorr, and Hyperscore. The RAId aPS implemented scoring functions performs comparably to the original implementation in other search methods. (PDF) [file pone.0015438.s010.pdf]

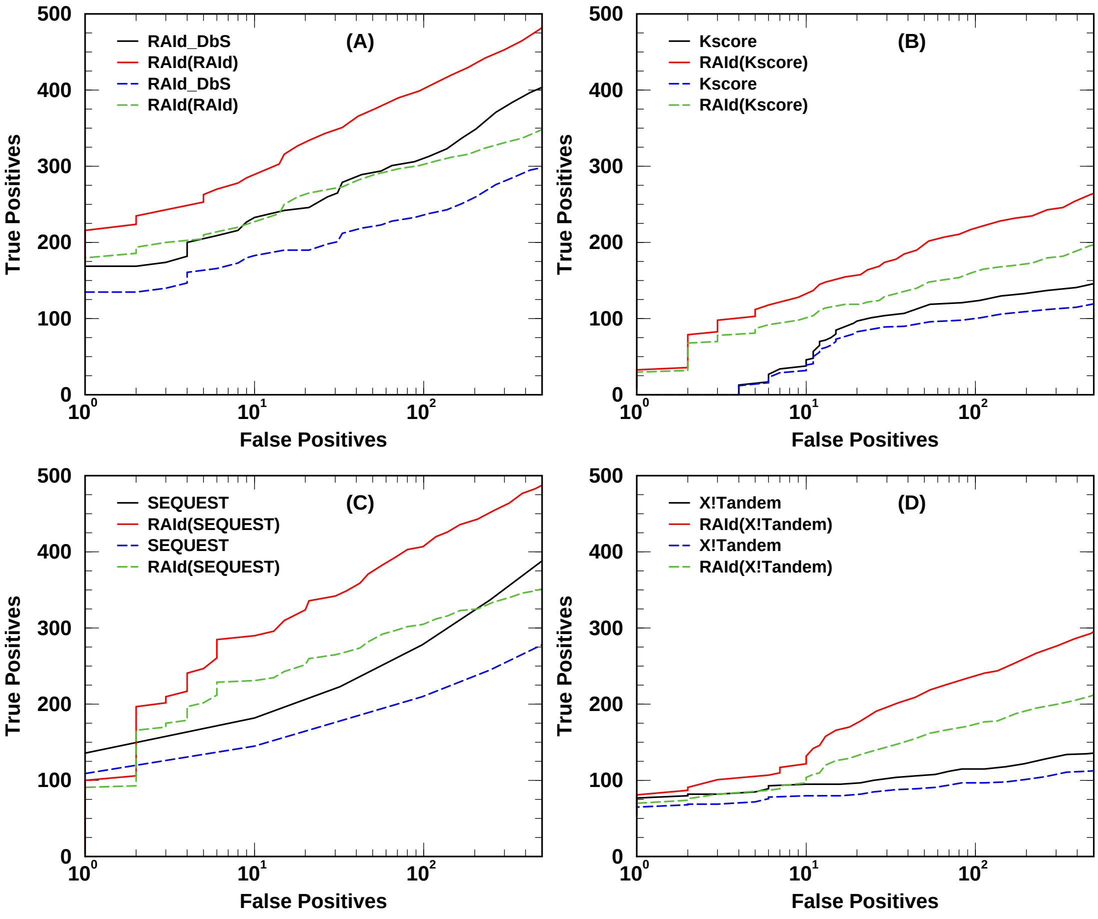

Figure S9. ROC curves when highly homologous proteins [1] are removed from the nr database and thus are not counted towards true positives or false positives. Each panel displays the results of a scoring function. The resulting ROC curves from using RAId_aPS implementation and the original implementation in other search program are both shown. The results from profile data (NHLBI data set [2]) are shown in solid curves, while the results from centroid data (A1-A4 of ISB data set [3]) are shown in long-dash curves. Panels (A,B,C,D) respectively display the results from using RAId score, K-score, XCorr, and Hyperscore. The RAId_aPS implemented scoring functions performs comparably to the original implementation in other search methods.

References

1. Alves G, Ogurtsov AY, Yu YK (2007) RAId_DbS: Peptide identification using database searches with realistic statistics. Biology Direct 2: 25.

2. Alves G, Ogurtsov AY, Wu WW, Wang G, Shen RF, et al. (2007) Calibrating E-values for MS^2 library search methods. Biology Direct 2: 26.

3. Keller A, Samuel P, Nesvizhskii AI, Stolyar S, Goodlett DR, et al. (2002) Experimental protein mixture for validating tandem mass spectral analysis. OMICS 6: 207-212.
